# Hypobaric hypoxia induced renal damage is mediated by altering redox pathway

**DOI:** 10.1371/journal.pone.0195701

**Published:** 2018-07-13

**Authors:** Varun Chhabra, Avnika Singh Anand, Amit Kumar Baidya, Shajer Manzoor Malik, Ekta Kohli, Maramreddy Prasanna Kumar Reddy

**Affiliations:** 1 Cardio-Respiratory Division, Defence Institute of Physiology and Allied Sciences (DIPAS), Defence Research and Development Organisation (DRDO), Ministry of Defence, Timarpur, Delhi, India; 2 Neurobiology Division, Defence Institute of Physiology and Allied Sciences (DIPAS), Defence Research and Development Organisation (DRDO), Ministry of Defence, Timarpur, Delhi, India; National Cancer Institute, UNITED STATES

## Abstract

Systemic hypobaric hypoxia is reported to cause renal damage; nevertheless the exact pathophysiological mechanisms are not completely understood. Therefore, the present study aims to explore renal pathophysiology by using proteomics approach under hypobaric hypoxia. Six to eight week old male Sprague Dawley rats were exposed to hypobaric hypoxia equivalent to altitude of 7628 metres (pO_2_-282mmhg) at 28°C and 55% humidity in decompression chamber for different time intervals; 1, 3, and7 days. Various physiological, proteomic and bioinformatic studies were carried out to examine the effect of chronic hypobaric hypoxia on kidney. Our data demonstrated mild to moderate degenerative tubular changes, altered renal function, injury biomarkers and systolic blood pressure with increase in duration of hypobaric hypoxia exposure. Renal proteomic analysis showed 38 differential expressed spots, out of which 25 spots were down regulated and 13 were up regulated in 7 dayhypobarichypoxic exposure group of rats as compared to normoxia control. Identified proteins were involved in specific molecular changes pertinent to endogenous redox pathways, cellular integrity and energy metabolism. The study provides an empirical evidence of renal homeostasis under hypobaric hypoxia by investigating both physiological and proteomics changes. The identification of explicit key proteins provides a valuable clue about redox signalling mediated renal damage under hypobaric hypoxia.

## Introduction

Hypoxia occurs when tissue supply of oxygen is decreased and it is encountered in many pathophysiological conditions, such as atherosclerosis, obstructive sleep apnea, mountain sickness, ischemic diseases, cancer and during embryonic development. Moreover, hypoxia is a very important factor affecting the health and life activities of individuals at high altitude, which has serious impacts on physiology and induces pathological changes in the body [1,2]. Further, several high altitude studies speculate that there is an increased risk for the development of kidney dysfunction characterized by micro albuminuria, hypertension, and hyper uricemia with relatively preserved glomerular filtration rate (GFR), known as high altitude renal syndrome (HARS) [3,4].

The increase in ventilatory response and diuresis during acute high altitude exposure appears to be an obligatory early phase of acclimatization to altitude. Moreover, volume regulation is considered one of the key physiological process central for both high altitude acclimatization, adaptation and mal-adaptation [5]. The strong positive correlation between the progression of kidney injury and decline in oxygen tension (pO_2_) suggests the existence of causal involvement between hypoxemia and renal dysfunction [6,7]. Additionally, many pathological conditions including chronic respiratory insufficiency, prolonged cardiovascular dysfunction, acute diabetes, hypertension, aging, renal hypertrophy, anemia and obstructive uropathy reduce renal oxygenation and results in kidney dysfunction [8]. Together, the available clinical and experimental evidences strongly indicate that kidney is vulnerable to hypoxia and play a very important role in high altitude adaptation.

Hypoxia has been implicated in both acute and chronic kidney injury; however the precise mechanisms underlying hypoxia-induced kidney injury remains poorly understood [8]. Very limited information is available on short term and long-term effects of hypobaric hypoxia on kidney structures and function. Further, the risk of high altitude-induced kidney dysfunction and its systemic effects are also currently unclear. Except few case reports, there is not enough evidence (human or animal studies) that proves an association between altered kidneys function and increased incidence of high altitude induced systemic complications [5,9].

Recent advances in proteomic techniques make it possible to monitor altered protein expression profile and may provide a better insight into the mechanisms involved in functional adaptations of cells, tissues, organs and the whole organism in extreme environment. Proteomic approach further helps in deciphering the alterations that occur in tissue specific proteins involved in health and disease and further enables identification of early diagnostic and therapeutic targets [10]. Similar approaches have previously been used for better understanding the underlying mechanism of renal toxicity and injury [11]. There is little or no information on the molecular mechanisms underlying hypobaric hypoxia-associated kidney damage and is indeed necessary to understand the molecular mechanisms contributing for the kidney dysfunction. These studies will facilitate the interventions to prevent the onset, progression and/or aggravation of kidney injury in high altitude sojourners as well as in high altitude native population. Therefore, the present study aims to address the structural, functional and molecular changes in rat kidney during exposure to chronic hypobaric hypoxia.

## Materials and methods

### Ethical approval

All protocols involving animal studies were reviewed and approved by the Institutional Animal Ethics Committee (IAEC) accredited to Committee for the Purpose of Control and Supervision of Experiments on Animals (CPCSEA), Government of India. All animal experiments were conducted in accordance with the National Institutes of Health (NIH) Guide for the Care and Use of Laboratory Animals. Rats were housed in cages under temperature, light and humidity controlled environment in institute (DIPAS) animal care facility. Animals were allowed to have free access to feed and water *ad libitum*. All the experimental procedures were followed as per standard experimental procedures to minimize the suffering of animals.

### Chemicals

All general chemicals utilized in the experiments were purchased from Sigma- Sigma Aldrich, St. Louis MO, USA, unless otherwise stated specifically. Kits, Primary antibodies and secondary antibodies were procured from multiple commercial suppliers as mentioned within the protocol. iSTATCG4+ cartridges were purchased from Abbott, East Windsor, N. J., USA.

### Experimental design

Twenty male Sprague Dawley weighing 250–260g were obtained from our Institute Experimental Animal facility, DIPAS, New Delhi and kept in our institute animal house at an ambient temperature of 25±2°C, with a 12 hour diurnal cycle. Standard chow and sterile water was available to rats *ad libitum*. Hypobaric hypoxia (HH) was induced in a specially design decompression chamber (Seven Star, India) as described elsewhere [12]. The male Sprague Dawley rats weighing 250–260g were subjected to simulated altitude of 25,000 feet (~7628 m) (pO_2_-282mmhg). Hypobaric chamber was maintained at ambient air temperature of 28°C and humidity 55%. Rats were divided into four groups (n = 5/group); 1 day, 3 day and 7 day hypobaric hypoxia group and the fourth group served as normoxia control group, maintained in similar cages at normal atmospheric pressure. Rats were allowed to free access to food and water during the exposure periods. The rate of ascent and decent was maintained at 300m/sec. The animals were kept at 12:12 hrs of light-dark cycles in a chamber. The chamber was opened for 15–20 minutes for replenishment of food and water on alternative days. Provision was made in the chamber for continuous replenishment of air. We did not notice any adverse clinical signs during exposure to hypobaric hypoxia nevertheless animals displayed lethargy and significant weight loss **(S1 Fig).**

### Systolic blood pressure measurement

In all experimental groups, systolic blood pressure (SBP) was measured using tail cuff plethysmography (NIBP, AD Instruments, Australia). All animals were preconditioned one week before taking the blood pressure (BP) measurements. Animals were kept in restrainers and three successive BP recordings were taken from each animal in an interval of 5 minutes. The SBP was monitored at baseline (day 0) and followed by various time points after the hypobaric hypoxia exposure (1, 3 and 7 days).

### Blood, urine collection and tissue preparation for histological studies

Blood samples were obtained by cardiac puncture using heparanized syringe under anesthesia urethane (1. 2g/kg of body weight, i. p) and 2 ml of blood was collected. Plasma was separated by centrifuging the blood at 2500 rpm for 20 min at room temperature. Urine samples were collected manually in Petri-dishes from rats after appropriate intervals of hypoxia exposure and Normoxia Control groups. Samples were immediately centrifuged (300 × *g*, 5 min) to remove debris or casts before storage. Plasma and urine samples were stored at -80°C until further analysis. After blood collection animals were euthanized with overdose of Sodium Pentobarbital (120mg/kg body weight, i.p). Then immediately, whole body vascular perfusion to remove residual blood from the tissues was done through left ventricle with ice cold normal phosphate buffer saline (1X PBS). Following perfusion, kidneys were collected. One kidney was kept in 10% formalin at 4°C for histological studies. The other kidney was immediately snap frozen and kept at -80°C until use for molecular studies.

### Renal function test and urinary protein concentration

Plasma Creatinine, Blood urea nitrogen (BUN), Urea and Uric acid were measured using relevant kits (Randox Laboratories, UK) according to manufacturer’s protocols. Urine protein measurement was done using Bradford assay kit (Sigma Aldrich, St. Louis, MO, USA), according to manufacturers’ protocol. The urine protein concentration was expressed in mg/ml.

### Histopathology

Kidney tissues stored in formalin were embedded in paraffin and 5 μm thick tissue sections were obtained. Sections were placed on frosted glass slides, stained with haematoxylin and eosin (H&E), Masson’s Trichome (MT), Sirius red (SR) and Periodic acid-Schiff (PAS). Histological changes in sections from rats exposed to different intervals (1, 3 and & 7 day) of hypobaric hypoxic and normoxia control rats were examined comparably. All sections were analysed by pathologist in a blind fold manner to evaluate vascular, glomerular, and tubular pathology of various experimental groups.

### Immunohistochemistry

The formalin fixed kidneys were cryoprotected by subjecting to serial dilutions of 10%, 20% and 30% of sucrose solution and finally embedded in Optimal Cutting Temperature (OCT)compound before tissues stored at -80° C. Then kidney tissue embedded in OCT compound were sectioned with 30μm size using cryostat (Lecia Bio systems, Germany). Cryosections of kidney tissue (normoxia and hypoxic groups) were washed with PBST containing triton X-100 and antigen retrieval was done with trypsin EDTA. After antigen retrieval, endogenous peroxide blocking was done with 0.3% hydrogen peroxide and followed by either 5% normal goat serum or fetal calf serum. After blocking, sections were permeabilized with 0.1% triton X-100 and incubated with respective primary antibodies for HIF-1 alpha (Sigma Aldrich, St. Louis MO, USA), Fibronectin (Sigma Aldrich, St. Louis MO, USA), CD14 (Santa Cruz Biotechnology Inc., USA), Collagen-1 (Sigma Aldrich, St. Louis MO, USA) and KIM-1 (Abcam, Cambridge, U. K), overnight in 1:250–500 dilution. After primary antibody incubation, sections were thoroughly washed with PBS containing 0.1% triton X-100 and the washed sections were further incubated with secondary antibody in 1:2000 dilutions for 2 hours. Then, sections were finally washed with PBS containing 0.1% triton X-100 and incubated in Diamino-benzidine (DAB; 1mg/ml) containing 0.1% peroxide in PBS.

After colour development, sections were carefully transferred on to the slides, spread and subjected to dehydration with 70%, 90%, and 100% alcohol. Section were counterstained with haematoxylin and mounted with DPX medium. Images were captured using Olympus BX51TF microscope, Japan at 40X magnification. For immunohistochemistry, experimenter calculated out the mean intensity of sections via image J,NIH, of normoxia and 7 day hypobaric hypoxia group of rats. The calculated mean intensity was further plotted into Graph Pad Software, California, USA.

### ELISA (enzyme-linked immunosorbent assay)

ELISA was carried out to measure plasma Cystatin-C (Santa Cruz Biotechnology, USA), Urine Netrin-1 (Bioss, USA), Urine IL-18 (Santa Cruz Biotechnology, USA). Urine KIM-1 (Abcam, Cambridge, U. K), Kidney ICAM-1 (Santa Cruz Biotechnology, USA), KidneyVCAM-1 (Santa Cruz Biotechnology, USA) in rats exposed to hypobaric hypoxia at different time points (1, 3 and 7 day) and in normoxia group. Protocol details including preparation of standard reagents were followed according to general protocols for indirect ELISA by Abcam (Cambridge, MA, USA; http://www.abcam.com/protocols/indirect ELISA protocol). 50 μg protein from plasma was coated onto 96 well plate overnight, then blocked with 3% BSA solution for 1hour, then primary antibodies for Cystatin-C (1:1000), Netrin-1 (1:500), IL-18 (1:1000). KIM-1 (1:1000), ICAM-1 (1:1000) and VCAM-1 (1:1000) were incubated overnight and then washed 3 times with PBS. A specific isotypic secondary antibody conjugated with enzyme (1:2000) dilution was incubated for 2 hrs. After completion of secondary antibody incubation, washing was done with PBST 4 times and then colour was developed using o-phenyl diamine (OPD) substrate. Intensity of colour (yellow) was measured at 450 nm using spectrophotometer and data obtained was normalised and plotted on Graph Pad Software, California, U. S. A.

### TUNEL assay

Tubular epithelial cellular apoptosis was examined by the TUNEL assay using TdTApopTag ® *In Situ* Apoptosis Detection Kit (S7165), Merck-Millipore (Merck-Millipore, USA) according to manufacturer instructions. Apoptosis (TUNEL assay) was compared between normoxia control and 7 day hypoxia group. The slides were counterstained with DAPI and mounted with mounting medium containing anti fade. Red colour TUNEL-positive nuclei were identified by using an Olympus BX51TF fluorescence microscope, Japan at 40X magnification equipped with a digital camera. For quantification, the number of positively red stained nuclei per field was determined and an average of ten randomly selected fields per animal was used for analysis. Results have been represented in terms of % positive nuclei.

### Protein extraction for proteomic studies

Frozen rat kidney was transversely cut from same position from all the normoxia and hypobaric exposure groups (7 day), in order to consider cortex as well as medulla. The tissues were weighed up to 100mg, chopped and homogenized using tissue homogenizer in lysis buffer containing 8M urea, 2M thiourea, 4% CHAPS, 40mM Tris, 1% DTT, 0.05% SDS and 1μl protease inhibitor cocktail for 10 min on ice. The tissue samples were then transferred in 1. 5ml micro-centrifuge tubes and centrifuged at 13,000 rpm for 15minutes at 4°C. The supernatant was then transferred into new tube and samples were stored in -20°C for further TCA/ Acetone precipitation. 20% TCA was added drop wise to one volume of protein sample (1:1) and mixed through vortexing and incubated for 1hour at -20°C. Samples were then centrifuged at 15000xg for 15 minutes at 4°C. After centrifugation, supernatant was taken and 0.5ml of ice-cold acetone containing 20mM DTT was added to wash the pellet. Samples were further centrifuged and acetone containing supernatant was removed. This acetone washing was repeated 2 times till the pellet turns to be white in colour and the pellet was air dried. For 2D gel electrophoresis, the protein pellet was suspended in 100μl lysis buffer, sonicated for 10 minutes for proper dispersion. The uniformly dissolved and distributed protein suspension was stored in -20° C until further analysis [13].

### First dimensional IEF using protean IEF cell

Protein concentration was estimated by Standard Bradford Method. A mixture of total 125μl rehydration buffer containing 8M urea, 2% CHAPS, 0.2% ampholyte, 50mM DTT, 0.001% bromophenol blue and 10μg protein was prepared. Immobilised linear pH gradient strips (IPG strips 7cm, pH 3–10,Biorad) were incubated in focusing tray with 125μl of rehydration buffer containing 10μg protein sample and subjected for automated passive rehydration for 14 hrs in protean IEF cell (Bio-Rad). IEF was conducted in protean IEF cell (Bio-Rad) at 20°C as follows: 200 V rapid for 2 hrs, 300 V gradual for 30 min, 1000 V rapid for 30 min, 5000 V rapid for 90 min, 5000 V linear for 1 hr and finally 500V on hold [13]. After focussing, strips were removed carefully and stored at -80° C until run on second dimension.

### Second dimensional electrophoresis and image analysis

For 2-DE analysis, individual samples (n = 5) from both the normoxia control and 7 day hypoxia exposure group were repeated in duplicates. Prior to SDS-PAGE, the IPG strips were equilibrated twice for 15 minutes with gentle shaking. The first equilibration solution containing 50 mM Tris-HCL, pH 8.8, 6 M urea, 30% glycerol (v/v), 2% SDS (w/v), 1% DTT (w/v). In the second equilibration buffer DTT was replaced with 2.5% iodoacetamide. The equilibrated IPG strips were slightly rinsed with milli-Q water, to remove excess equilibration buffer. Strips were applied to precast gradient gels (4–20%) and 7cm Mini Protean TGX (BIORAD Lab). The gels were run using a PROTEAN System (BIORAD Lab) at 100V until the dye front run off the edge of the 2D gel. After electrophoresis, proteins were visualised in the gel by silver staining procedure. The silver stain gels were scanned using an automated scanner and digitized images were analyzed using DELTA 2D Software. Automatic spots detection, spots matching and spot alignment in the gels was done, followed by manual rechecking of matched and unmatched spots. The intensity volumes of individual spots were normalized with the total intensity volume of all the spots present in the gel (%V). The principle of measuring intensity values by 2D analysis software is similar to densitometry measurement. Detected protein spots showing a change of more than 1.2 fold (p<. 005) between different samples groups were considered further analysis by MALDI.

### In gel digestion

In brief, protein spots were extensively washed with ultrapure water and each gel spot was precisely excised with a scalpel blade. The excised spots were de-stained and incubated for 30 min with 30mM potassium ferric-cyanide and 100mM sodium thiosulphate at room temperature. The gel pieces were rinsed several times with water to remove de-staining solution. The gel pieces were washed with water and 50mM ammonium bicarbonate/ acetonitrile. Enough acetonitrile was added to cover gel pieces for shrinking the gel pieces. The gel pieces were rehydrated in 10mM ammonium bicarbonate for 5 minutes, equal volume of acetonitrile was added and removed after 15 min of incubation. The gel pieces were again covered with acetonitrile and removed. The gel pieces were digested with 20μl of trypsin, sequencing grade (Promega Corporation, USA) and incubated the sample at 37° C overnight followed by sonication of tryptic peptides for 10min. The peptides were extracted with 0.1%TFA and dried in a speed vac.

### MALDI TOF/TOF

The peptides for each spot extracted with 0.1%TFA were spotted on MALDI sample target plate and mixed with equal volumes of α-cyano-4-hydroxy-cinnamic acid matrix solution (10mg/ml) prepared 50% acetonitrile with 0.1% TFA. The MS and MS/MS of peptide were acquired on a 5800 MALDI-TOF mass spectrometer (Applied Bio systems) operating on reflectron positive ion mode. Laser intensity was set at 3400 for MS and 3900 for MS/MS with 1000 and 3000 shots per spectrum respectively over a window MS of m/z 700 to m/z 4000. A combined MS peptide and MS/MS peptide sequencing search was performed against NCBI database against *Rattus via* Mascot search engine v2. 2. The search parameters allowed oxidation of methionine as variable modification, carbamido methylation of cysteine as fixed modification and a single missed tryptic cleavage. The mono isotopic precursor ion tolerance was set 250ppm and MS/MS ion tolerance to 0.4Da; the protein identification was accepted with a statistically significant probability based MASCOT score (p ≤ 0.05) **(S1 File)**. Proteins with at least peptides identified were reported to ensure accuracy of the identification.

### LC MS

Peptides were solubilised in 2% acetonitrile (ACN) (Sigma Aldrich, St. Louis, MO, USA) with 0.1% formic acid (FA)Sigma Aldrich, St. Louis, MO, USA). Samples were analyzed on a TripleTOF5600 (Sciex, USA) system coupled to an EksigentNanoLC-Ultra 2Dplus system. Peptides were desalted and concentrated for4 min on a C18 trap column, then separated on a Pepmap C18 reversed phase column (LC Packings) by elution with an ACN gradient Solvent B (5% to 90*% v/v ACN)* at a flow-rate of 0.7 μL min^−1^ for a time of 4 minutes. Finally, the column was re-equilibrated by solvent A (100% water, 0.1% FA) for 9 minutes. A total time of 22 minutes for completion of one sample. Positive electrospray ionization was then utilized as the ionization source. MS analysis was performed using a continuous duty cycle of survey MS scan followed by up to MS/MS analyses of abundant peptides. For data acquisition ranges were 100-1600m/z for MS and between 100- 1800m/z for MS/MS. The data files were processed by ProteinPilot 4.0 (AppliedBioSystems) using the Paragon algorithm. All searches were performed in the UniProt database (Swiss-Prot database) against Rattus Species with one missed cleavage, carboxymethyl modification of cysteines was a fixed modification and methionine oxidation was selected as a variable modification. Proteins with at least peptides identified with 1% FDR were reported to ensure accuracy of the identification.

### Bio-informatics analysis

Gene ontology based classification of identified proteins was done using Panther software 10.0.On the basis of their involvement, identified proteins were classified according to molecular function, biological process and cellular component. Network analysis of identified proteins was done using STRINGS 10.0 (http://www.stringsdb.co.in) with a confidence level of 0.4 with not more than 20 interactions [14].

### Western blotting

Tissues were lysed in urea buffer (7M urea, 2M thiourea and 4% CHAPS) containing protease inhibitor cocktail and the concentration of the protein was determined by the standard Bradford method. Equal amount of proteins (40 μg) was separated on 12–15% SDS-PAGE and transferred to PVDF membrane. After transfer, gel profile on the membrane was visualised with Ponceau Sto check equal loading [15]. Membranes were blocked using 3% BSA and incubated with Superoxide dismutase 1 (Santa Cruz Biotechnology Inc., USA), cytoplasmic 1 actin (Sigma Aldrich, St. Louis MO, USA) at room temperature for2 hrs. Subsequently, membranes were washed with PBS—0.1% Tween-20 (PBST) thrice for 5 minutes and were then incubated with their secondary antibodies conjugated with HRP for 2 hrs at room temperature. The membrane was washed thrice for 5 minutes with PBST and protein bands were visualised by enhanced chemiluminescence (ECL, Amersham Biosciences, Bucks, UK) in UVP Bio-Spectrum Imaging System and the densitometry analysis was performed with Image J software (http://rsbweb.nih.gov/ij/).

### Immunofluorescence

Cryostat sections were blocked with 3%serum and labelled with anti-Cu/Zn SOD (SOD1) (1:1000; Santa Cruz Biotechnology Inc., USA), for 2hrsat room temperature, followed by Alexa Fluor 488-conjugated anti-goatIgG antibody (1:2000; Invitrogen, California, USA) for 60 min at room temperature. Sections were counterstained with DAPI (Sigma Aldrich, St. Louis, MO, USA) and took images using the Olympus BX51TF fluorescence microscope, Japan at 40X magnification equipped with a digital camera. To analyse the expression of immunofluorescence data, experimenter calculated out the mean intensity of sections via image J of Normoxia and 7 day hypobaric hypoxia group of rats. The calculated mean intensity was further plotted into Graph Pad Software, California, USA.

### Biochemical assays

Biochemical assays were performed between normoxia control and 7 day hypobaric group of rat to confirm oxidative stress and energy status in kidney tissue. GST activity measurement and ATP levels were done by using GST and ATP kit (Sigma Aldrich, St. Louis, MO, USA) as per manufacturer instructions respectively. While ROS levels were measured according to DCFHDA method [16] and SOD activity according to pyrogallol method [17].

### Blood gasometry and clinical biochemistry analysis

i-STAT analyser and i- STAT cartridge CG4+ (Abbott, East Windsor, N. J. USA) were used to measure blood gasometry and acid–base balance between 7 day hypobaric hypoxia group and normoxia control group. Following parameters like blood pH, blood gas composition (pCO_2_- partial pressure of Carbon Dioxide, pO_2_- partial pressure of oxygen, S_v_O_2_ –percentage saturation of oxygen in mixed venous blood) and blood electrolyte (Base Excess, Lactate, HCO_3_^−^ –bicarbonate, Na^+^ –ionized sodium). Utmost care was taken to avoid blood hemolysis and coagulation while sample drawing and loading oniSTAT cartridges for analysis.

### Statistical analysis

All data were expressed as Mean ± Standard error of mean (S.E.M). Statistical analysis of data was done with the help of Graph Pad Software, California, U. S. A. Comparison between multiple groups (normoxia control, 1day, 3day and 7 day hypoxia) were analysed by One Way ANOVA followed by Bonferroni/Dunn method for multiple group comparison and unpaired t-test is to compare data between two groups (i.e. normoxia control and 7day hypoxia). Significant difference was considered if *P* <0.05.

## Results

### Effect of chronic hypobaric hypoxia on renal structure

**Fig 1:**
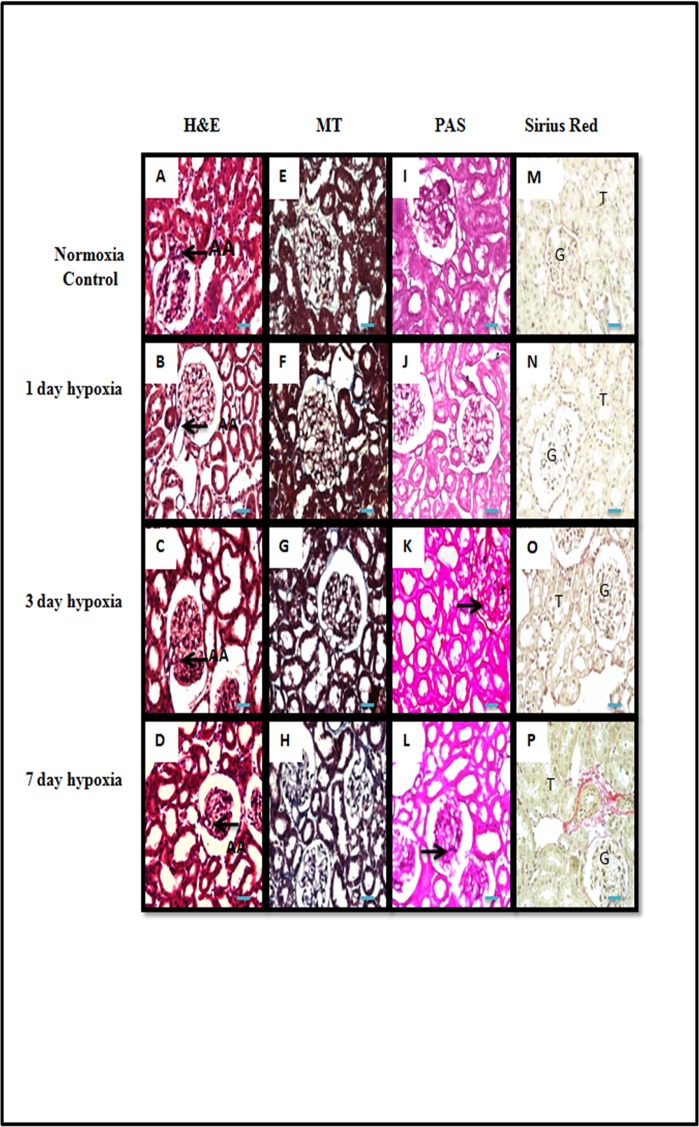
Representative pictomicrograph of rat kidney cortical tissue sections of normoxia control and in different durations of hypobaric hypoxia exposure. (A-D), Haematoxylin & Eosin Staining: Renal cortical sections showing characteristic histological signs of injury with afferent arteriolopathy, mild to moderate tubular degeneration, tubular dilation (thinning of tubular epithelium) and leukocyte infiltration of tubulointerstitium in all hypoxia exposure group as compared to normoxia control group of rats. (E-H) and (M-P), Masson’s Trichome and Picrosirius Red staining: representative pictures showing an increase interstitial collagen deposition in 3^rd^ day and 7 day hypobaric hypoxia exposure group of rats. (I-L), Periodic Acid Schiff staining (PAS): representative pictures showing enhanced mesangial proliferation and glomerular basement membrane thickening observed in 3 and 7 day hypobaric hypoxia exposure group of rats (n = 5). G = Glomerulus, AA = Afferent Arteriole, T = tubular epithelial cells. Scale bar refers to 100μm at 400x.

To understand the effect of hypobaric hypoxia, we carefully examined the kidney tissue sectioned from rats exposed to different durations (1, 3 and 7 day) of hypobaric hypoxia and normoxia control groups. In H&E staining, we found afferent arteriolopathy, leukocyte infiltration and thinning of tubular epithelium in hypoxic group of rats (Fig 1A–1D). These changes started appearing from 1^st^ day of hypoxia exposure and progressively increased by the 7^th^ day of hypobaric hypoxia exposure. We also found mild deposition of collagen in tubulointerstitial and perivascular area in 3^rd^ day and 7^th^ day hypobaric hypoxic group of rats which was shown in Masson‘s Trichome (Fig 1E–1H) and Picrosirius red stained sections (Fig 1M–1P). Similarly, we also found modest mesangial proliferation and basement membrane thickening in 3^rd^ and 7^th^day hypoxic group of rats as shown in Periodic Acid Schiff Staining (PAS) (Fig 1I–1L).

### Effect of hypobaric hypoxia on kidney function and systolic blood pressure

**Fig 2:**
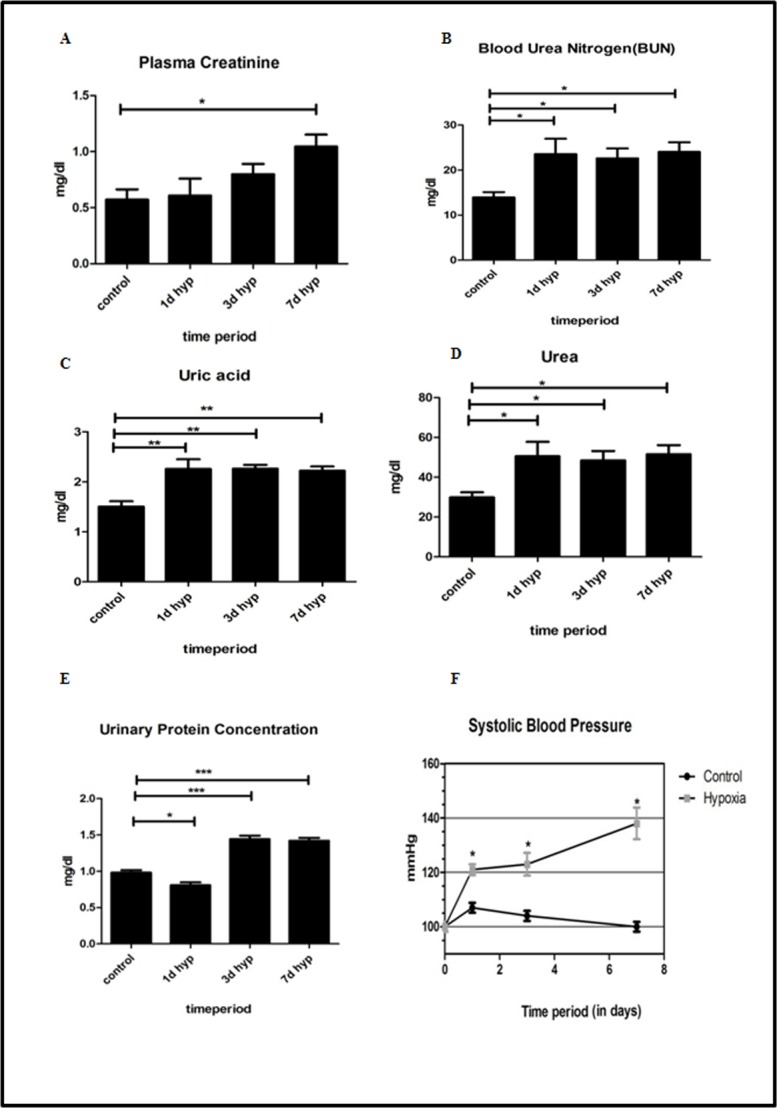
Effect of hypobaric hypoxia on kidney function. Biochemical and hemodynamic parameters estimated in control (normoxia) and experimental groups (different intervals of hypobaric hypoxia exposure) (n = 5). (A) Plasma Creatinine. (B) Blood urea nitrogen (BUN). (C) Uric acid. (D) Urea. (E) Urinary Protein concentration. (F) Systolic blood pressure. Data represented here is Mean ± S. E. M. Values are significant if *P*< 0.05. *stands for level of significance when *P*<0.05, **when *P*<0.05, *** when *P*<0.05 vs. control.

Plasmacreatinine levels were measured according to Jaffe’s method. Plasma creatinine levels were remained unaffected on day 1, started increasing from 3^rd^day of hypobaric hypoxia exposure and a significant increase (*P*<0.05) was found on 7^th^ day of hypoxia exposure (Fig 2A). Blood urea nitrogen levels were significantly (*P*<0.05) increased in all groups of hypoxic animals (1, 3and 7day) as compared to normoxia group of rats (Fig 2B). Urea levels were significantly (*P*<0.05) increased in all groups of hypoxic animals (1,3 and 7 day) as compared to normoxia group of rats (Fig 2D).

Uric acid levels were significantly increased (*P*<0.05) in all hypoxic groups of animals. The maximum increase in uric acid level was found in 7^th^ day hypoxic group when compared to normoxia control group of rats (Fig 2C). Initially, urinary protein concentration was significantly decreased on day 1 hypobaric hypoxia group. However, the protein concentration in urine was significantly increased (*P*<0.05) in day 3 and day 7 hypoxia exposure group (Fig 2E) when compared to normoxia group of rats.

We further sought to determine the effect of hypobaric hypoxia on systemic blood pressure. We noticed a progressive and significant increase (*P*<0.05) in systolic blood pressure in rats subjected to hypobaric hypoxia for different time points (1,3 and 7^th^ day). In our studies, the maximum increase in systolic blood pressure was seen on 7^th^ day of hypoxia exposure group (Fig 2F) when compared to normoxia group of rats.

### Hypobaric hypoxia induced renal insult and early biomarkers

**Fig 3:**
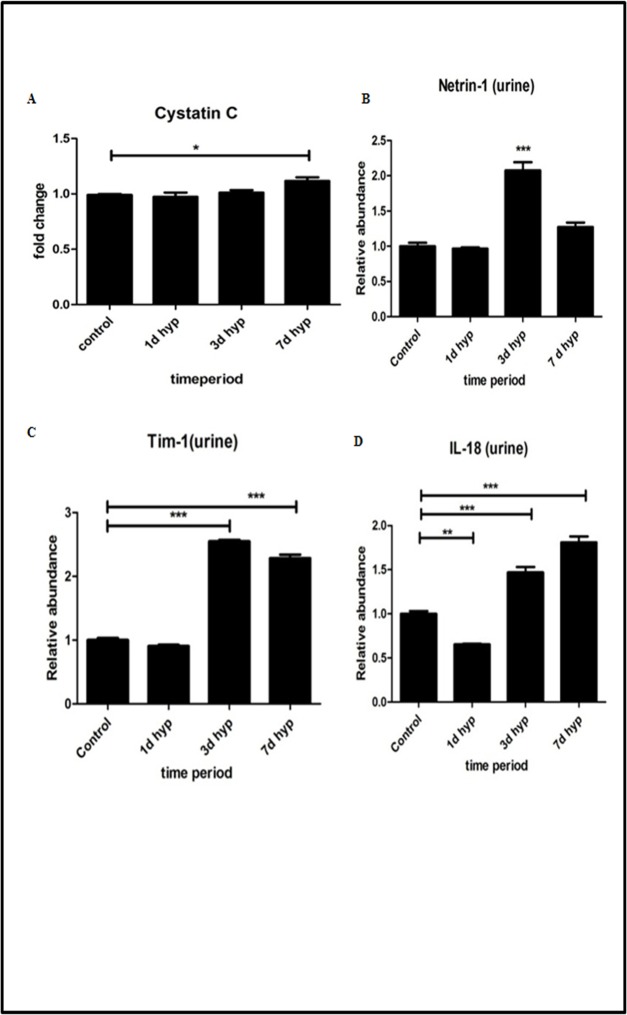
Effect of hypobaric hypoxia on kidney injury biomarkers. (A) Cystatin C in plasma. (B)Netrin-1 in urine. (C) Kim-1 in urine. (D) IL-18 in urine. Data represented here is Mean ± S. E. M. Values are significant if *P*<0.05. * stands for level of significance when *P*<0.05, **when *P*<0.05, *** when *P*<0.05 vs. control.

Cystatin C is a novel marker for kidney dysfunction. It levels was measured in rat plasma samples by Indirect ELISA method. Results were expressed in terms of fold change. Cystatin C levels were found to be normal in day 1 hypoxia group, whereas its levels gradually increased from day 3 and found to be maximum increase (*P*<0.05) on 7^th^ day of hypoxia exposure (Fig 3A) when compared to normoxia control group of rat. Netrin-1 is a novel marker of kidney injury. Its level was measured in rat urine samples by Indirect ELISA method and values were expressed in terms of fold change (Fig 3B). Netrin -1 levels were found to be normal in day 1 hypoxia group and gradually increased by 3^rd^ day of hypoxia. However, on 7^th^ day of hypoxia exposure, its levels were decreased when compared to 3 day hypoxia group, but found to be higher than the normoxia group.

Kim-1 is also a novel marker of acute kidney injury. Its level was measured in rat urine samples by indirect ELISA method and values were expressed in terms of fold change (Fig 3C). Its levels were found to be significantly increased on 3^rd^ day of hypobaric hypoxia with maximum observed rise in 7^th^ day hypobaric hypoxia exposure group of rats compared to normoxia group. Urinary IL-18 has been detected in patients suffering from acute kidney injury and is another novel marker of acute kidney injury. Its levels were measured in rat’s urine samples by indirect ELISA method and values were expressed in terms of fold change (Fig 3D). Its levels were found to be significantly increased (*P*<0.05) on 3^rd^ day of hypobaric hypoxia with maximum increase on 7^th^ day of hypobaric hypoxia exposure.

**Fig 4:**
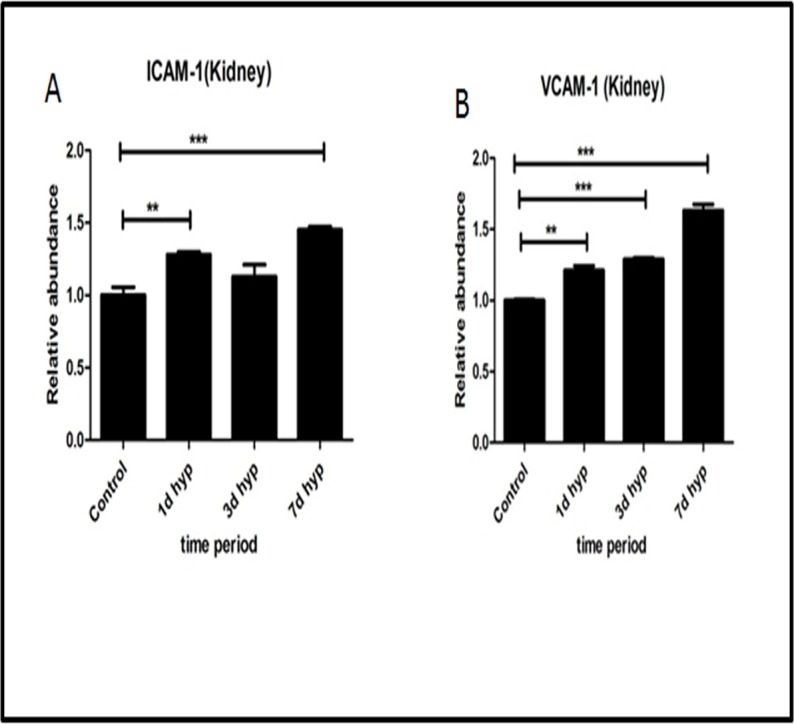
Effect of hypobaric hypoxia on inflammatory cell adhesion molecules. (A)ICAM -1 (in kidney). (B) VCAM-1 (in kidney). Data represented here is Mean ± S. E. M. Values are significant if *P*<. 05. * stands for level of significance when *P*<0.05, **when *P*<0.05, *** when *P*<0.05 vs. Control.

Vascular cell adhesion molecules (VCAM-1) and Intracellular cell adhesion molecules (ICAM-1) are the cell adhesion molecules expressed on endothelium, which are up regulated in inflammation. We measured VCAM-1 and ICAM-1 levels in rat kidney tissue homogenates by indirect ELISA method. Results were expressed in terms of % fold change in O. D values. Our results demonstrated a significant increase of adhesion molecules (VCAM-1 and ICAM-1) in rats exposed to hypobaric hypoxia and we observed a maximum rise in these adhesion molecules in rats exposed to 7 days hypobaric hypoxia (Fig 4A and 4B).

### Hypobaric hypoxia induced progression of kidney injury

**Fig 5:**
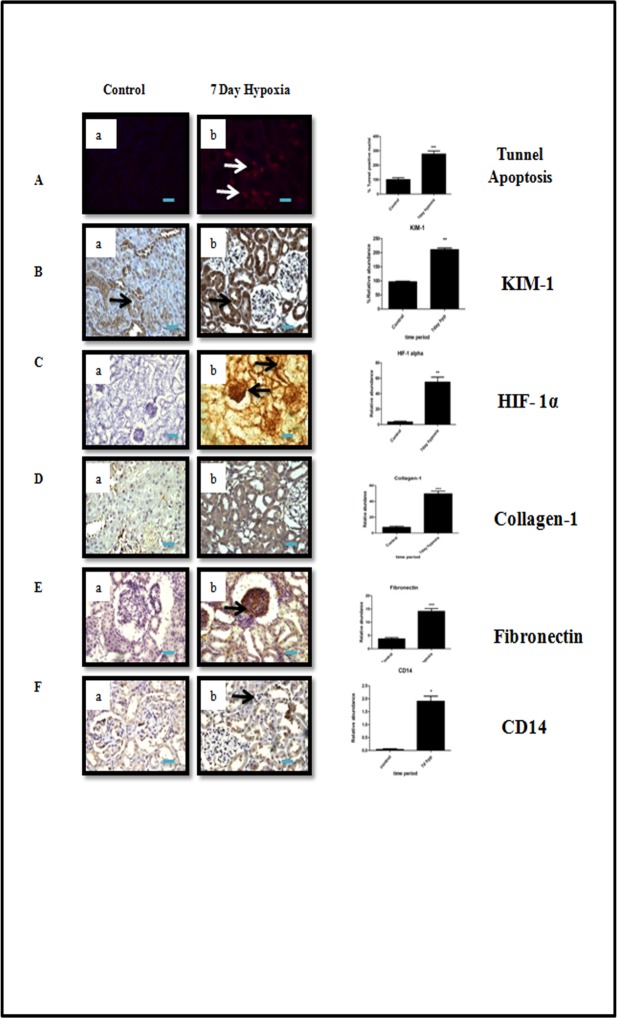
Immunohistochemical analysis of tubulointerstitial injury and other cellular changes under hypobaric hypoxia. (A) Tubular apoptosis by tunnel method. (B) Renal injury by Kim-1. (C) Hypoxia response by HIF-1alpha. (D-E) Fibrosis: Collagen-1 and Fibronectin. (F) Macrophage infiltration CD14. Data represented here is Mean ± S. E. M. Values are significant if *P*<. 05. * stands for level of significance when *P*<0.05, **when *P*<0.05, *** when *P*<0.05 vs. control. Scale bar refers to 100μm at 400x and 200μm at 200x.

We found a significant increase in number of tunnel positive nuclei (*P*<0.05) in tubular epithelial cells on 7^th^day of hypobaric hypoxia exposed group of rats when compared to normoxia group (Fig 5A) indicating apoptosis could be a possible trigger for activation of subsequent pathways involved in the progression of kidney injury. Supporting above assumption, we also found a significant increase in Kim-1 expression in rats exposed to 7 days of hypobaric hypoxia as compared to normoxia group of rats (Fig 5B). Hypoxia Inducible factor 1 alpha (HIF1α) is generally up regulated in response to hypoxia. We measured HIF1α levels in normoxia control as well as in 7 day hypobaric hypoxia exposure group of rats by immunohistochemistry. The HIF1α levels were significantly (*P*<0.05) increased in 7 day hypoxia group of rats when compared to normoxia control group. However, we noticed that HIF1α was mainly localized in proximal, distal and collecting ducts (Fig 5C). Collagen-1 and Fibronectin were reported to be associated with the activation of profibrotic response in kidney [7]. Our study showed a significant increase (*P*<0.05) in the expression of fibronectin in focal glomeruli and tubulointerstitial (cortex) region in rats exposed to 7 day hypobaric hypoxia when compared to normoxia group of rats. (Fig 5D and 5E).

It is well established that mononuclear phagocytes, including macrophages, play a central role in inflammatory diseases. In addition, hypoxia has also been reported as one of the factors responsible for inflammation. We found a significant increase (*P*<0.05) in immune reactive CD14 macrophage cells in renal cortex of 7 day (Fig 5F).

### Chronic hypobaric hypoxia induced changes in renal proteomic profile

**Fig 6:**
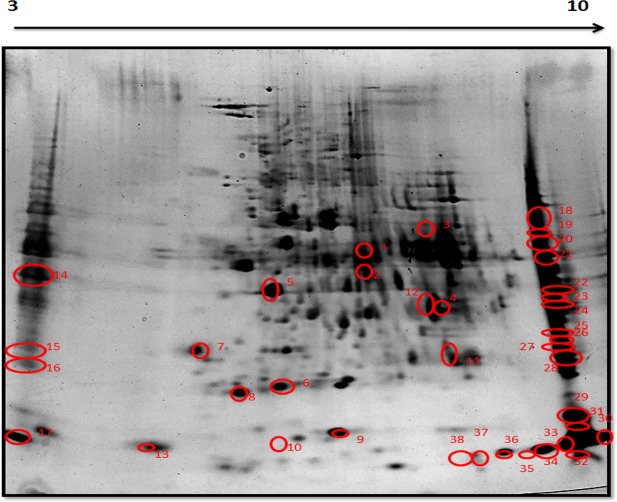
Representative image of master gel showing differentially expressed spots in 2DEfrom rat kidney tissues. Firstly, the proteins were resolved according to their Isoelectric point (pI) in the (3–10 pH) and then separated according to their MW on 4–15%gradient SDS-PAGE gradient gel followed by silver staining. Numbers marked on protein spots were differentially expressed in 7 day hypoxia exposed group.

**Fig 7:**
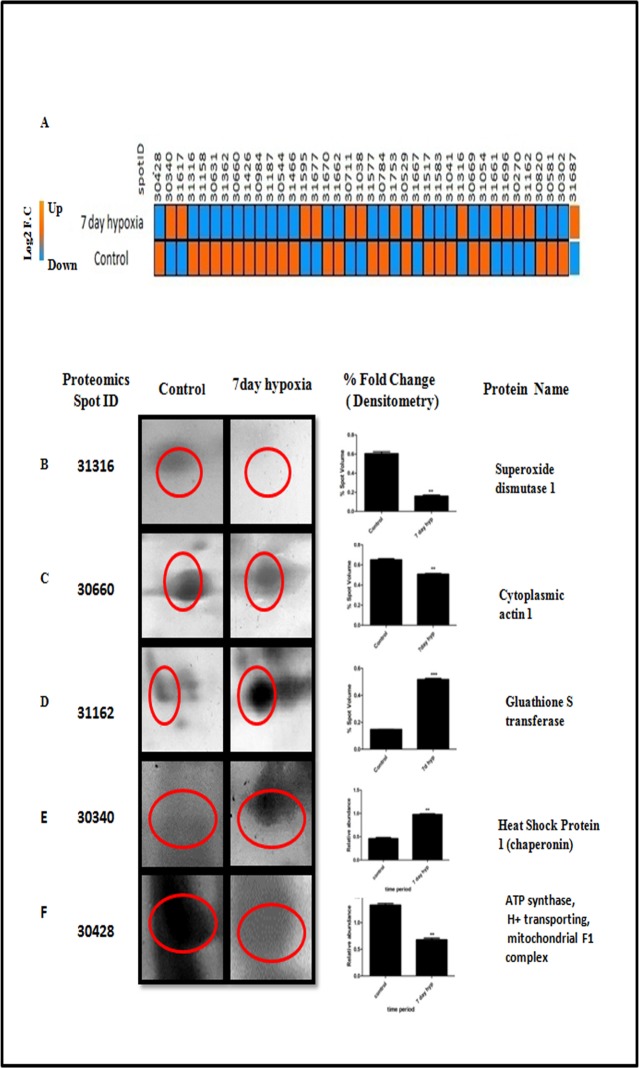
**A. Heat map showing differently expressed spots obtained after 2DE**. Spots that showed up regulation in hypobaric hypoxia condition shown in orange. While spots that showed down regulation shown in blue. Fig 7 (B-F). Magnified comparison maps of spot 31316, 30660 31162, 30340 and 30428 in the 2DE patterns of control and 7 day hypoxia. Spot 31316, 30660 and 30428 had decreased expression in the 7 day hypobaric hypoxia exposure group while 31162 and 30340 had increased expression in 7 day hypoxia group.

A total of 1400 spots were detected and matched between the 7 day hypobaric hypoxia group vs. normoxia control group of rats on 2D gel electrophoresis images. The master gel showed a total of 38 differentially expressed spots, out of which 25 spots were down regulated and 13 spots were found to be up regulated (Fig 6, Fig 7A and Table 1). The cut off value at 1.2 fold increase or decrease was considered for significant change. Out of 38 differentially expressed spots, 32 spots were further processed for identification by MALDI MS/MS analysis and LCMS analysis (Table 2, Table 3).

**Table 1:** List of differently expressed spots with proteomics spot ID and their respective fold changes.

S. No.	Spot No.	Proteomics Spot ID	Protein name	Fold Change^#^
1	19	30428	ATP synthase, H+ transporting, mitochondrial F1 complex	-1.8
2	1	30340	Heat shock protein 1 (chaperonin)	1.90
3	10	31617	Clathrin heavy chain linker domain-containing protein 1	9.17
4	6	31316	Cu-Zn Superoixde Dismutase	-4.13
5	27	31158	eukaryotic translation initiation factor 4 gamma 3	-4.21
6	21	30631	hypoxia up-regulated protein 1 precursor, cytochrome b-c1 complex subunit 2, mitochondrial precursor	-1.37
7	8	31362	Similar to microfilament and actin filament cross linker protein isoform	-1.24
8	5	30660	Actin, cytoplasmic 1	-1.28
9	28	31426	Peptidyl‐prolyl cis‐trans isomerase A	-2.28
10	24	30984	Voltage‐dependent anion‐selective channel protein 1	-1.35
11	26	31187	Glutathione S‐transferase alpha‐3	-2.4
12	14	30544	1 (peptides not Matched)	-1.4
13	29	31466	cytochrome c, somatic	-2.71
14	9	31595	2 peptide not matched	2.42
15	36	31677	Haemoglobin alpha chain	7.19
16	34	31670	Haemoglobin Beta chain	-1.5
17	33	31662	Hemoglobin subunit beta‐1	-1.78
18	12	30711	T cell receptor V delta 6, beta-arrestin-2	2.88
19	15	31038	3 (Peptides Not Matched)	1.20
20	30	31577	ATP synthase, H+ transporting, mitochondrial F1 complex, epsilon subunit	-5.37
21	4	30784	Alcohol dehydrogenase [NADP+]	-7.28
22	32	31753	10 kDa heat shock protein	4.2
23	20	30529	ATP synthase subunit alpha, mitochondrial	-1.2
24	35	31667	Hemoglobin subunit alpha‐1/2	1.27
25	13	31517	alpha-2u globulin	-9.78
26	17	31583	Heat-responsive protein 12	-3.07
27	25	31041	4 (Peptides Not Matched)	-1.52
28	16	31177	5 (peptide not matched)	1.55
29	22	30669	Isocitrate dehydrogenase [NADP], mitochondrial	-1.67
30	7	31054	6 (Peptides Not Matched)	-2.19
31	31	31661	rCG34342, isoform CRA_b	5.31
32	38	31696	beta-globin [Rattus norvegicus]	31.01
33	3	30274	E3 ubiquitin-protein ligase TTC3	3.57
34	11	31162	Glutathione–S–Transferase P	3.55
35	23	30820	Aldolase B	-2.33
36	2	30581	7 (Peptides Not Matched)	-1.22
37	18	30302	8 (Peptides Not Matched)	-1.93
38	37	31687	Hemoglobin subunit alpha‐1/2	13.11

Note:-

‘**#**’ indicates *P* value less than 0.05 as compared to control group.

**Table 2:** List of differently expressed spots during hypobaric hypoxia exposure, identified by MALDI-TOF/TOF.

S. No.	Spot Id	Protein Name	Expected pI/MW	Gene Accession ID	Mascot Score	Species	Peptide sequence	Fold change^#^
1	30820	Aldolase B	3.67 /40035	gi|1619606	77	Rattus	K.WRAVLR.I	-2.332
2	31316	Cu-Zn Superoixde Dismutase	4.95 /16155	gi|155369696,	46	Rattus	R.MIQRLR.S	-4.2
3	30784	Alcohol dehydrogenase [NADP+]	6.84/ 36711	gi|13591894	189	Rattus	R.SPAQILLR.W K.YALSVGYR.H	-7.28
4	31162	Glutathione–S–Transferase P	9.02 / 23652	gi|25453420	72	Rattus	M.PPYTIVYFPVR.G	3.55
5	30711	T cell receptor V delta 6,	8.84/31399	gi|6724195	36	Rattus	SGRYSVVFQK SLK	2.88
6	30274	E3 ubiquitin-protein ligase TTC3	6.11/225.79	NP001101785	42	Rattus	R.HLCQPR.G	3.57
7	30428	ATP synthase, H+ transporting, mitochondrial F1 complex	7.04 / 5528.94	gi|149029483	88	Rattus	K.GPVGSKIR.R	-1.88
8	30660	Actin, cytoplasmic 1	5.54 / 42.25	gi|4501885	143	Rattus	K.RGILTLK.Y	-1.287
9	31362	Similar to microfilament and actin filament cross linker protein isoform	5.5/612088	gi 149023883	49	Rattus	R.INQLSAR.W R.NLQDSIK.R	-1.2
10	31426	Peptidyl‐prolyl cis‐trans isomerase A	8.34/18091	PPIA_RAT	41	Rattus	K.SIYGEKFEDENFILK.H	-2.28
11	30984	Voltage‐dependent anion‐selective channel protein 1	8.62/30.8	VDAC1_RAT	88	Rattus	R.EHINLGCDVDFDIAGPSIR.G	-1.35
12	31187	Glutathione S‐transferase alpha‐3	8.78/25360	GSTA3_RAT	42	Rattus	M.PGKPVLHYFDGR.G	-2.43
13	31677	Haemoglobin alpha chain	7.82/15490	HBA_RAT	164	Rattus	K.LHVDPENFR.L	7.191
14	31670	Haemoglobin subunit beta	7.88/16083	HBB1_RAT	150	Rattus	K.IGGHGGEYGEEALQR.M	-1.50
15	31662	Hemoglobin subunit Beta	7.88/16083	HBA_RAT	174	Rattus	K.IGGHGGEYGEEALQR.M	-1.77
16	31577	Cytochrome c, somatic	9.61/11712	CYC_RAT	64	Rattus	K.TGPNLHGLFGR.K	-5.3
17	31753	10 kDa heat shock protein	8.89/10895	CH10_RAT	73	Rattus	K.VVLDDKDYFLFR.D	4.25
18	30529	ATP synthase subunit alpha, mitochondrial	9.2/59831	ATPA_RAT	51	Rattus	R.TGAIVDVPVGDELLGR.V	-1.26
19	30669	Isocitrate dehydrogenase [NADP], mitochondrial	8.88/51391	IDHP_RAT	62	Rattus	R.GKLDGNQDLIR.F	-1.677
20	31667	Hemoglobin subunit alpha‐1/2	7.82/15490	HBA_RAT	107	Rattus	K.IGGHGGEYGEEALQR.M	1.27
21	31687	Hemoglobin subunit alpha‐1/2	7.82/15490	HBA_RAT	108	Rattus	K.IGGHGGEYGEEALQR.M	13.11
22	31661	rCG34342, isoform CRA_b	6. 64/15490	EDM04010.1	48	Rattus	K.IGGHGGEYGEEALQR.M	5.319

Note:-

‘**#**’indicates *P* value less than 0.05 as compared to control group.

**Table 3:** List of differently expressed spots during hypobaric hypoxia exposure, identified by LC MS.

S. No	Spot ID	Name	Gene accession ID	Sequence Coverage	Peptide 95%	Species	Fold change^#^
**1**	30631	hypoxia up-regulated protein 1 precursor, cytochrome b-c1 complex subunit 2, mitochondrial precursor	gi|77404380, gi|55741544	7.4, 48.5	1,9	Rattus norvegicus	-1.374
**2**	31158	eukaryotic translation initiation factor 4 gamma 3	gi|157817837	3.3	1	Rattus norvegicus	-4.211
**3**	31466	cytochrome c, somatic	gi|6978725	95.2	29	Rattus norvegicus	-2.713
**4**	31577	ATP synthase, H+ transporting, mitochondrial F1 complex, epsilon subunit	gi|34786049	15.7	1	Rattus norvegicus	-5.377
**5**	31696	beta-globin	gi|56252	34	1	Rattus norvegicus	31.01
**6**	31583	Heat-responsive protein 12	gi|51259281	71.5	8	Rattus norvegicus	-3.076
**7**	31677	beta-globin [Rattus norvegicus]	gi|984679	40	1	Rattus norvegicus	7.191
**8**	31617	Clathrin heavy chain linker domain-containing protein 1	gi|81883834	3.4	1	Rattus norvegicus	9.1
**9**	31517	alpha-2u globulin	gi|8307686	85.1	33	Rattus norvegicus	-9.7
**10**	30340	Heat shock protein 1 (chaperonin)	gi|55778012	4.2	1	Rattus norvegicus	1.9
**11**	30711	beta-arrestin-2	gi|6978539	3.4	1	Rattus norvegicus	2.88

Note:-

‘**#**’indicates *P* value less than 0.05 as compared to control group.

Some of the identified proteins were classified into following categories: 1) Cellular enzymes related to cell redox homeostasis and oxidative stress, under which our results demonstrated upregulation of glutathione S transferase protein (GSTP1) and downregulation of Cu-Zn SOD (Fig 7B and 7D), 2) Proteins related to structural integrity, under which our results demonstrated downregulation of cytoplasmic actin protein (Fig 7C) and upregulation of spectrin beta non erthyocytic protein, 3) Metabolism related enzymes, in this category; we observed that aldolase b and ATP Synthase F1 H+ transporting alpha subunit (Fig 7F)were downregulated and alcohol dehydrogenase was upregulated in 7 day hypobaric hypoxia exposedgroup,4) Proteins related to inflammation such as T cell receptor V delta 6 was found to be upregulated in hypobaric hypoxia group, 5) Protein belonging to ubiquitin proteosomal degradation pathway, anE3 ubiquitin protein ligase TTC3 was upregulated in hypobaric hypoxia group.

### Molecular pathways involved in hypobaric hypoxia induced kidney damage

**Fig 8:**
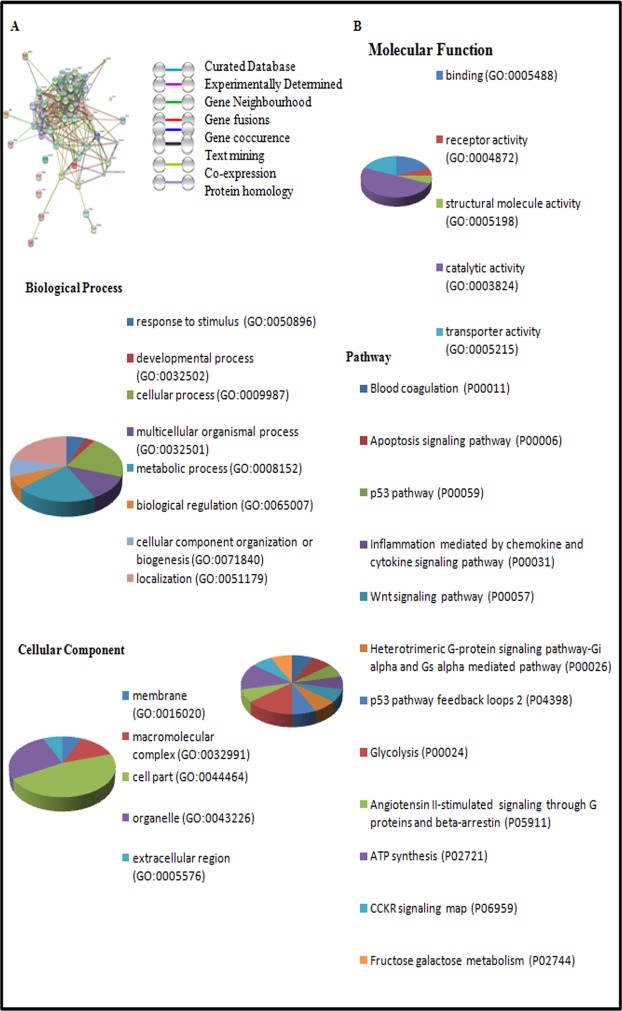
Network analysis and gene ontology annotations of the proteins identified by MALDI-TOF MS. **A)** Network analysis of identified proteins with their predicted interactions using STRING db 10.0 software. **B)** Gene ontology classifications (Pie chart analysis) of proteins were obtained by using PANTHER 10.0 software and proteins were distributed according to their Biological Process, Molecular Function, Cellular Component and Pathway Involved.

The known and predicted interactions of detected proteins in GO classification with other proteins were obtained by searching the STRING db online web server 10.0. Search was performed at confidence level of 0.4 with not more than 20 interactions based on neighbourhood, gene fusion, co-occurrence, co-expression, experimental, databases, text mining and homology (Fig 8A). Based on gene ontology (GO) analysis (Fig 8), the identified proteins were classified into 1) Biological process, (Fig 8B), 2) Molecular function (Fig 8B). 3) Cellular component (Fig 8B). The most predominant annotated class of the biological process are a) Response to stimulus, b) Developmental process, c) Cellular process, d) Biological regulation, e) Cellular component organisation, f) Localisation g) Metabolic Process h) Multicellular Organismal Process (Fig 8B). The most predominant annotated class of molecular function are: a) Binding, b) receptor activity c) Structural molecular activity, d) Catalytic activity, e) Transporter activity (Fig 8B). The most predominant annotated class of cellular component are a) Membrane, b) Macromolecular complex, c) Cell part, d) Organelle e) Extracellular region (Fig 8B).

The most predominant Pathways are: a) Blood Coagulation; b) apoptosis Signalling pathway; c) p53 pathway; d) inflammation mediated by chemokine and cytokine signalling pathway; d) Wnt signalling Pathway; e) Heteromeric G Protein Signalling pathway; f) Glycolysis; g) p53 pathway feedback loop 2; h) Angiotensin ii stimulated Signalling through G Proteins and Beta arrest in; i) ATP synthesis; j) CCKR signalling; k) Fructose galactose metabolism (Fig 8B).

Based on KEGG pathway enrichment analysis (Table 4) and Panther pathway. analysis, some of major pathways which were affected by these identified proteins were as follows (Table 4). I) ATP metabolic process. II) Oxidative phosphorylation. III) Glycolysis and Gluconeogenesis; IV) Removal of Superoxide Radicals; V) Hypoxia Response; VI) Glutathione Metabolism; VII) Regulation of actin cytoskeleton.

**Table 4:** List of pathways obtained by KEGG pathway enrichment analysis of some of these identified proteins.

S. No.	Pathway involved	FDR^#^ (False Discovery Rate) P-value	Proteins involved
1.	ATP metabolic process	3.30E-26	Atp5a1, Aldob
2.	Oxidative phosphorylation	1.37E-28	Atp5a1
3	Glycolysis / Gluconeogenesis	1.04E-23	Akr1a1, Aldob
4	Glutathione metabolism	0.000231	Gstp1
5	Regulation of actin cytoskeleton	0.0383	Actb,
6	Carbon metabolism	1.13E-21	Aldob
7	Metabolism of xenobiotics by cytochrome P450	1.17E-10	Gstp1
8	Phagosome	3.20E-08	Aldob
9.	Drug metabolism—cytochrome P450	1.55E-05	Gstp1
10	Removal of superoxide radicals	0.0145	Sod2, Sod3, Sod1
11	Response to reactive oxygen species	0.0306	Gstp1,
12	Response to hypoxia	0.0417	Sod2, sod1
13.	Transport	0.000241	Atp5a1
14	Response to stimulus	0.00044	Aldob, Gstp1,

Note:-

‘**#**’indicates *P* value less than 0.05 as compared to control group.

Diagrammatical representations of some of the major Pathways affected under hypobaric hypoxia can be obtained and viewed in online KEGG pathway mapping software [18]. (A) Glutathionemetabolism- (**http://www.genome.jp/kegg-bin/show_pathway?map=map00480&show_description=show-**). (B) Redox pathway- (**http://www.genome.jp/keggbin/show_pathway?map=hsa04213&show_description=show-**) (C) Oxidative phosphorylation (**http://www.genome.jp/keggbin/show_pathway?map=map00190&show_description=show**) for ATP generation pathway affected under hypobaric hypoxia.

### Validation of proteomics data using immunoblot, immunofluorescence and biochemical assays

**Fig 9:**
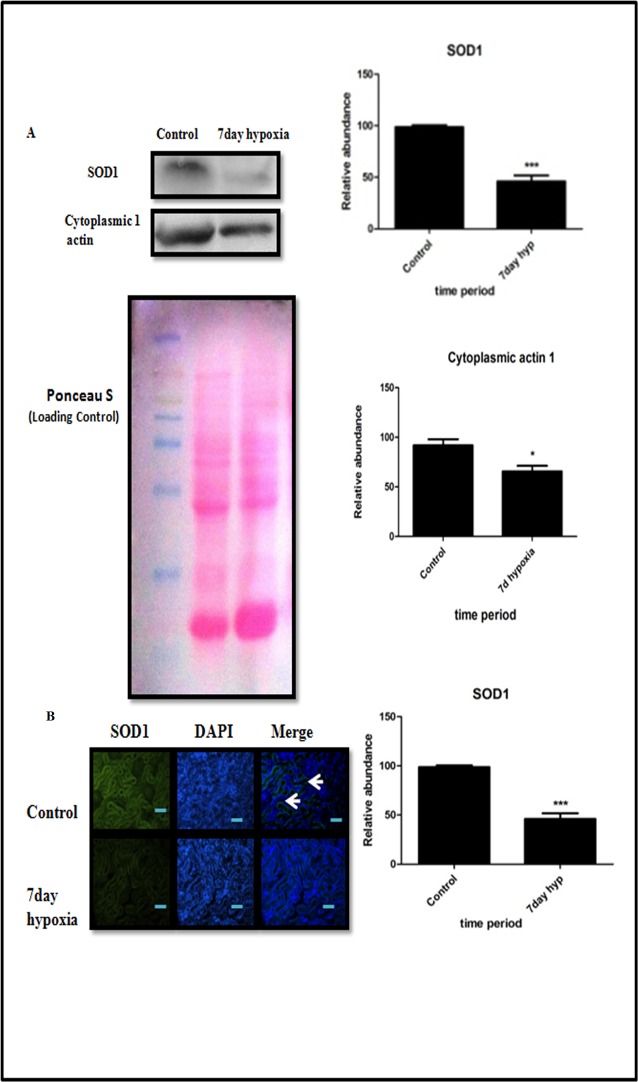
Validation of proteomic data by Immunoblottting and immunofluorescence. **(A)** Western blot analysis of SOD1 and cytoplasmic 1 actin of control and 7 day hypoxia group. Kidney tissue and their respective optical densities (ROD) and densitometry analysis of results from western Blot indicating significant change between two groups compared by Student ‘s t-test **(B)** Immunofluorescence analysis of SOD1 in control and 7 day hypoxia exposure group. Results have been represented in term of %. Values are significant if *P*<0.05. * stands for level of significance when *P*<0.05, **when *P*<0.05, *** when *P*<0.05 vs. control.

**Fig 10:**
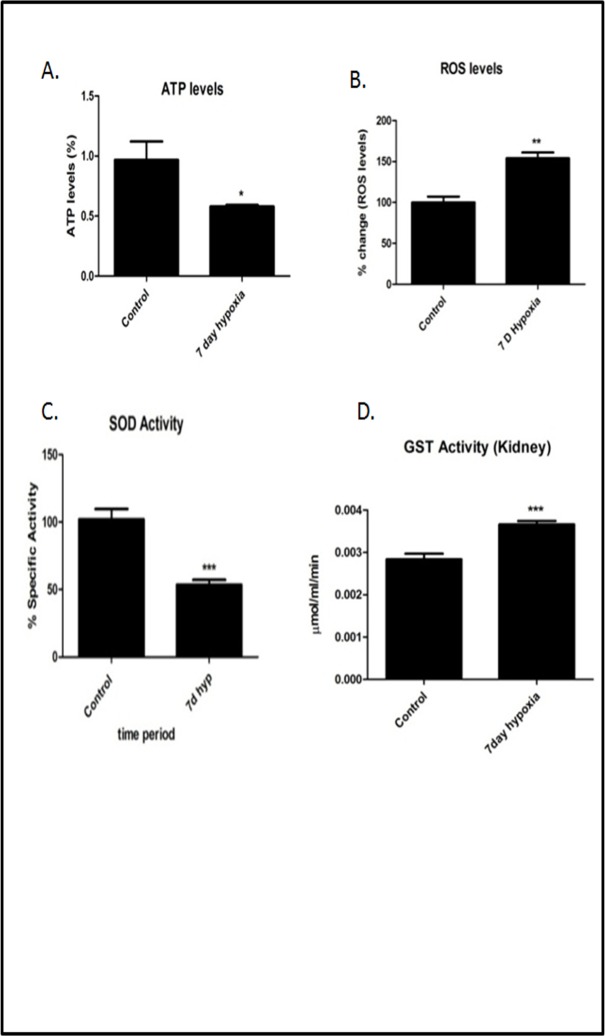
Biochemical assays showing redox parameters and ATP levels in 7 day hypobaric hypoxia. A) ATP levels (B) ROS levels (C) SOD activity levels (D) GST activity levels. Results have been represented in term of %. Values are significant if *P*<0.05. * stands for level of significance when *P*<0.05, **when *P*<0.05, *** when *P*<0.05 vs. control.

Proteomic data was also confirmed by immuno blotting analysis that showed Superoxide Dismutase 1 and cytoplasmic actin were down regulated in7 day hypoxia exposed group of rats (Fig 9A). We further validated the expression of SOD1 by Immunofluorescence (Fig 9B)which was found to be significantly decreased in7 day hypoxia exposed rats. ATP levels, SOD activity and GST activity levels (Fig 10A, 10C and 10D) were also measured to validate proteomics data. ATP levels were significantly lower (*P*<0.05) (Fig 10A) and GST activity levels (Fig 10D) were significantly higher (*P*<0.05) in7 day hypobaric hypoxia exposure group respectively that is relatively consistent with proteomic data. We also measured ROS to measure oxidative stress occurring due to down regulation of Cu-Zn SOD (Fig 10B, Fig 10C).

### Alteration of blood gasometry, pH and electrolytes in hypobaric hypoxia

We made an attempt to monitor blood gases in rats during hypobaric hypoxia exposure, but we were not successful in drawing arterial blood under hypobaria. On the contrary, the pressure inside the chamber was taken to confirm the level of hypoxia (FiO2).

Ideal method for arterial blood gases analysis is to collect the arterial blood from aorta and carotid artery but we collected blood from cardiac puncture which contains both venous and arterial blood. We measured the same immediately after removing the rats from decompression chamber after 7 days of hypoxic exposure and the observed blood gas parameters (mixed venous blood) were incorporated in the study (Table 5).

**Table 5:** Effect of hypobaric hypoxia on pH, blood gas variables and blood electrolytes in 7 days hypobaric hypoxia exposed rats.

S. No.	Blood gas variables (Mixed venous blood)	Normoxia Control	7 day hypoxia#
**1.**	**pH**	7.371 ± 0.02520	7.157 ± 0.03251 **
**2.**	**pCO**_**2**_ **(mmhg)**	67.13 ± 4.886	42.72 ± 2.044 **
**3.**	**pO**_**2**_ **(mmhg)**	40.67 ± 0.3333	26.60 ± 3.945*
**4.**	**Base Excess (mmol/L)**	0.6667 ± 2.404	-12.33 ± 2.092 **
**5.**	**HCO**_**3**_^**-**^ **(mmol/L)**	26.80 ± 2.822	16.22 ± 1.598 **
**6.**	**T**_**CO2**_ **(mmhg)**	30.83 ± 3.941	17.50 ± 1.648 **
**7.**	**S**_**v**_**O**_**2**_ **(%)**	68.97 ± 1.742	44.00 ± 6.528 *
**8.**	**Lactate (mmol/L)**	1.360 ± 0.3233	8.354 ± 1.837 *

**‘#’** Data represented here Mean ± S. E. M. (n = 5). Values are significant if *P*<0.05.

* stands for level of significance when *P*<0.05

**when *P*<0.05

*** when *P*<0.05 vs. normoxia control.

There is a significant (*P*<0.05) decrease in blood pH, pO_2_, pCO_2_, base deficit, Tco_2_,HCO_3_^-^ and Sv_O2_ in 7 day hypobaric hypoxia group (Table 5). There is a significant increase (*P*<0.05) in lactate levels in the blood (Table 5). All these changes indicate that there was an increase in acidity in blood with decrease in oxygen saturation in hypobaric hypoxia group.

## Discussion

Using *in-vivo* physiological studies coupled with *proteomic* analysis, we evaluated the effect of hypobaric hypoxia on renal structure, function and proteomic profile in rat model. Our findings indicate that prolong hypobaric hypoxia causes kidney damage with significant structural and molecular changes pertinent to cell membrane integrity, endogenous redox pathway and energy metabolism. Unravelling these cellular and molecular mechanisms of hypobaric hypoxia induced renal changes may increase our understanding on high altitude acclimatization, adaptation and mal-adaptation [18]. Further, it may open new therapeutic modalities to minimize and/or prevent the hypobaric hypoxia induce renal damage and associated systemic effects.

There are very few morphological studies that address the progressive changes in the kidney during chronic exposure to hypobaric hypoxia for different intervals of time [19,20,21]. The present study demonstrated significant structural changes such as thickening of glomerular basement membrane, shrinking of afferent arterioles, degenerative changes in tubular epithelial cells and modest deposition of extracellular matrix proteins in tubule interstitium of rats subjected to sustained exposure to hypobaric hypoxia for different time points (1, 3 and 7 day). The observed hypobaric hypoxia induced structural changes in kidney may have an important role in high altitude renal syndrome (HARS) reported in some patients [3,6].

The available experimental evidences together with our results strongly indicate that the hypobaric hypoxia induced early structural changes in kidney may affect the kidney function and influence the different body systems [22]. Traditionally, the kidney function is determined byserumcreatinine and blood urea nitrogen levels [23]. Nevertheless, under hypobaria it needs to be confirmed as apparent differences in fluid flux has been reported due to barometric pressure difference. Further we are also aware of the limitations associated with traditional biomarkers that are influenced by several non-renal factors independent of kidney function [23] and the severity of kidney damage. Creatinine, blood urea nitrogen and urea for detection of kidney function, the abnormal creatinine levels are noticed only when 50–60% kidney function is lost. Further, creatinine release is influenced by age, gender, diet, muscle mass and vigorous exercise. Conversely, blood urea takes time to accumulate and poorly reflects real time changes in GFR [24].

Various animal model of hypoxia mediated renal injury like I/R renal injury, Renal artery Stenosis, hypobaric hypoxia model, demonstrated slight increase and/or near normal level of serum creatinine [19,25,26]. Our data also showed similar pattern of creatinine profile during different intervals of hypobaric hypoxia exposure, suggesting hypoxia induced kidney damage may not severe enough to reflect marked increase in creatinine levels. Also, the rise in BUN/Urea levels in our model is significantly less as compared to other animal renal injury like I/R renal injury, gentamicin, cisplatin, mercury chloride and contrast induced nephropathy [25,27,28,29]. The observed mild changes in both Plasma Creatinine and BUN/Urea in our study may partly be due to decreased blood volume, inter-compartmental fluid shift and acidemia which are cause due to acclimatization under hypoxic stress. Serum uric acid (UA), the final product of purine degradation and there is increase in adenine turnover, oxidative stress, polycythemia, increase lactate production and decrease urate secretion observed in hypoxia [19,30]. All these factors might be contributing together for elevation of uric acid in our study. There is also an increase prevalence of proteinuria in people living at high altitude. The pathogenesis of proteinuria depends on multiple factors like increased hematocrit, hypervicousity, elevated blood pressure, hyperuricemia and metabolic acidosis in hypoxic environment of renal parenchyma [30]. In our study, increased urinary protein concentration has been observed due to contribution of these multiple factors in hypoxia and suggestive of initiation of kidney damage.

However, our study perhaps not directly addresses the issue of evaluating the normal and abnormal values of these parameters under hypobaric conditions in order to make an adequate interpretation of these results and draw any valid conclusions. Nevertheless, our results strongly indicate that the sustained exposure to prolonged durations of hypobaric hypoxia would have inflected the permanent and irreversible loss of renal function with marked increase of above markers in the circulation. The conventional clinical biomarkers for renal dysfunction such as serum creatinine, BUN and estimated glomerular filtration rate, are inadequate in their ability to detect mild to moderate degree of kidney injury [31,32,33] and this notion may be supported by recent study where they reported increase in prevalence of unrecognized kidney disease in healthy people living at high altitude [34]. We therefore considered monitoring panel of novel kidney injury biomarkers such as KIM-1/TIM-1, Cystatin C, IL-18 and Netrin-1 along with traditional clinical measures to make adequate interpretation of our findings on renal function and draw reasonable conclusions of our finding on kidney injury and dysfunction during hypobaric hypoxia exposure. Currently, development and validation of high-throughput innovative technologies allow rapid multiplexed detection of multiple markers like Cystatin C, netrin-1, IL-18 and Kim-1 in the blood and urine which has more specificity and sensitivity to detect early changes of kidney damage and associated dysfunction [35,36,37]. In the present study, we evaluated the different established makers such as Cystatin C, Netrin-1, Kidney Injury Molecule-1 (KIM-1)/Tcell immunoglobulin and mucin domains-containingprotein-1 (TIM-1) and interleukin 18 (IL18) plasma and urine biomarkers, which could possibly help in detecting the early kidney injury during exposure to hypobaric hypoxia. Increased expression of these novel biomarkers in various durations of hypoxia provides valuable evidence of initiation of hypoxic renal injury.

ICAM-1 and VCAM-1 are cell adhesion molecules that belongs to immunoglobin superfamily members and have an important role in ischemia reperfusion injury by regulating leukocyte recruitment [38,39]. In our study, we have found that there is a significant increase in ICAM-1 and VCAM-1 levels in 3 and 7 day hypoxia which suggests that is an occurrence of leukocyte infiltration in hypoxic kidney.

The present study further demonstrated the progression of kidney injury by establishing the tissue specific pathological indicators, such as apoptosis of tubulare pithelial cells, HIF-1α, and pro-fibrotic markers Fibronectin, and collagen-1 and macrophage infiltration CD14. The increased expression of these pathological markers undoubtedly indicate that hypobarichypoxia exposure may cause initiation and progression of kidney injury similar to ischemia/reperfusion injury [19,40] [.

Acute Hypobaric hypoxia is associated with decrease in PO_2_, CO_2_ and Hco_3_- [41]. Another study showed a decrease in arterial pO_2_,pCo_2_, pH and HCO_3_- in rats exposed to chronic normobaric hypoxia [42]. In our study, we studied the direct impact of hypobaric hypoxia of 7 days in rats and found a significant decrease in PO_2_, CO_2_ and HCO_3_-. This states that decrease in environmental air pressure directly reduces oxygen saturation, CO_2_ concentration and HCO_3_- in blood. There is also fall in pH and significant increase in lactic acid in 7 day hypobaric group which indicating metabolic acidosis. Metabolic acidosis provides a valuable clue that there is increased in anaerobic respiration in renal tissue and muscle under hypobaric hypoxia. Metabolic acidosis also caused due to decrease in bicrobarbonate, ph Pco_2_ [43]. The ultimate consequences of this acidemia are weakness, muscle and kidney damage [43,44].

Currently, we don’t have any specific physiological and/or molecular biomarkers which could aid in differentiating high altitude acclimatized vs. non-acclimatized individuals [45]. Understanding the early kidney responses to acute hypobaric hypoxia and subsequent molecular mechanisms involved in progression of kidney injury may help in detecting susceptible individuals to high altitude maladies [46]. Recently many novel technologies (genomics, proteomics and metabolomics) have made it easier to understand pathophysiological mechanisms and identify the molecular signatures as potential biomarkers.

In the present study, we used gel based proteomic approach to examine the renal proteome and understand the complex process of hypobaric hypoxia induced molecular changes in kidney. We compare the renal tissue proteomic profiling of rats exposed to 7 days of hypobaric hypoxia with normoxia control group. We found 38 differently expressed protein spots in hypobaric hypoxia kidney as compared to normoxia control.

In our study, we observed one of the most remarkably changes in category of proteins of the family of Glutathione S-transferases (GST). The products of GST catalysis are more water-soluble, promoting ROS detoxification and thereby protecting tissues from oxidative damage. In mammals, GSTs are divided into 3 Super families: Cytosolic, Mitochondrial and Microsomal. Glutathione S-tranferase pi GSTP1 belongs to Cytosolic Super family [47] [. At present, eight distinct classes of the soluble cytoplasmic mammalian glutathione S-transferases have been identified: alpha, kappa, mu, omega, pi, sigma, theta and zeta. This gene encodes a Glutathione S-tranferase belonging to the alpha class genes that are located in a cluster mapped to chromosome 6 and are highly related with glutathione peroxidase activity. It has been known from several earlier studies that hypoxia causes oxidative stress [48]. It may be reasonable to assume that GST levels would be upregulated in order to increase the cellular capacity to restore redox homeostasis [47]. Corroborating the above assumption, our proteomic data confirmed the up regulation of GSTP1 protein activity in kidneys in rats exposed to hypobaric hypoxia compare to normoxia group. Glutathione S-transferase alpha-3 (GSTA3), a member of an important family of detoxifying and cytoprotective enzyme is reported to play a critical role in various diseases associated with alternation in oxidation-regulating proteins. Some studies reported that GSTA3 has been playing an important role in development of kidney fibrosis and liver fibrosis via mediating through oxidative stress pathway [49,50]. In our study, the observed decrease in expression of GSTA3 in 7 day hypobaric hypoxia exposure rat kidneys emphasizes its role in oxidative stress-induced kidney damage. Similarly, superoxide dismutase (SOD) belongs to another endogenous antioxidant enzyme family of proteins; primarily involved in converting superoxide radical to molecular oxygen and hydrogen peroxide. Based on cofactor they contain, these SODs are divided into 4 types. Iron SOD (Fe-SOD), manganese SOD (Mn-SOD), copper-zinc SOD (Cu-Zn SOD), and nickel SOD (Ni-SOD). Out of these 4 SODs, Cu-Zn SODs is the most abundant and found in cytosol and extracellular space. As reported in previous study, we found decreased expression of Cu-Zn SOD in hypobaric hypoxia exposed kidney, confirming the role of oxidative free radicals in hypoxia induced kidney damage [51]. Further, it has also been reported that intra-renal hypoxia per se, without confounding factors such as hyperglycemia and oxidative stress, can also induce nephropathy [8]. Moreover, it is also rational to believe that the increase in oxidative stress in renal tissue may augment the sympathetic activity and also influence the bioavailability of vasoactive mediators thus affecting the systemic circulation [8,52]. This was evident from our results, where the hypobaric hypoxia exposed rats showed significant elevation of systolic blood pressure.

Mitochondrial complex V or ATP Synthase is an enzyme complex that works as a molecular machine to generate and hydrolyze ATP in cells in the last step of the mitochondrial respiratory process. Consequently, ATP Synthase plays pivotal roles not only in maintaining the cellular energy state, but also in determining mitochondrial respiratory function. ATP Synthase is composed of two linked multi-subunit complexes; the soluble catalytic core (F_1_) and the membrane-spanning component (F_o_) comprising the proton channel. The Fo complex contains transmembrane subunits that transport protons from the inter membrane space and the F1 is a peripheral complex in the matrix, which catalyzes nucleotide binding for ATP synthesis [53]. Our results demonstrated downregulation of ATP Synthase H+ transporting F1 protein levels in hypobaric hypoxia group of rats as compared to normoxia control, indicating hypobaric hypoxia may cause decline in levels of ATP production. The inherent feature of low ATP production renal tissue compounded with hypoxia induced complex pathophysiological process sets in the kidney and may induce drastic decline in the availability of ATP for essential cellular functions [54]. Several experimental studies implicated the decrease in ATP levels as contributing factor for ischemic and hypoxic insults in kidney. In the line of previous studies, our results provide a valuable clue to believe that the gradual decline in ATP levels in the kidney during hypobaric hypoxia may be a reason for kidney insult.

Aldolase B plays a key role in carbohydrate metabolism as it catalyzes one of the major steps of the glycolytic-gluconeogenic pathway [55]. Aldolase B or liver-type Aldolase is one of three isoenzymes (A, B, and C) of the class I fructose1,6-bisphosphate aldolase enzyme (EC 4. 1. 2. 13) and plays a key role in both glycolysis and gluconeogenesis. The significant decrease in levels Aldolase B expression in hypobaric hypoxia group of rats indicates the shift in metabolic pathways.

**Fig 11:**
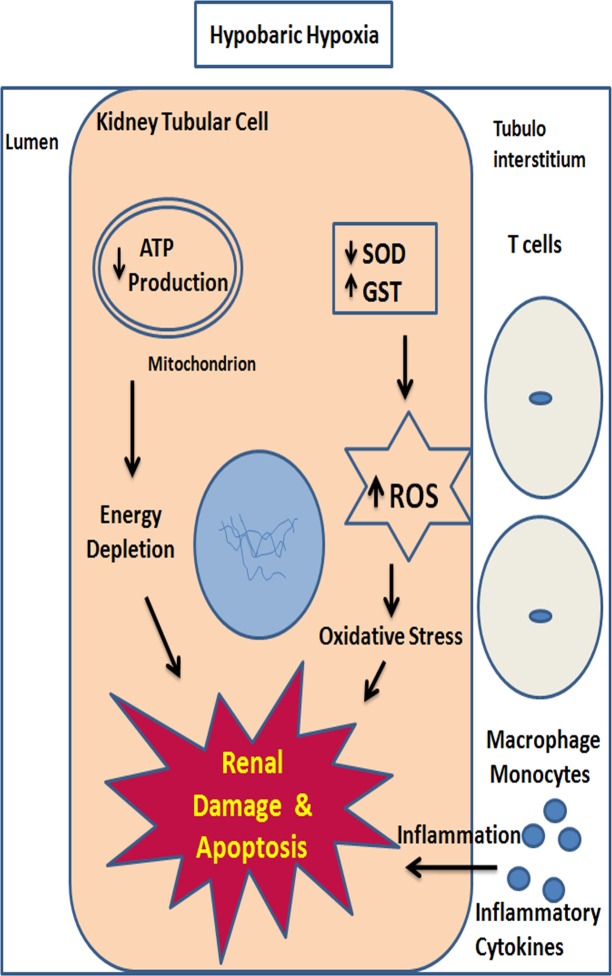
A hypothetical schematic diagram showing molecular events occurs during hypobaric hypoxia in kidney. Increased oxidative stress, inflammation and decreased ATP levels together contribute towards progression of renal injury in hypobaric hypoxia.

T cell receptor V delta 6, the part of adaptive immune system, is present on T cells. From the literature, it has been known that TCR’s (αβ and δγ) play a crucial role in promoting ischemia reperfusion injury [56]. In our study, we found an increased expression of TCR V delta 6, indicating its role in hypoxic induced inflammation and subsequent kidney injury. Additionally, in our study we have seen a decrease in levels of cytoplasmic actin protein, which directly indicates the disruption of cellular integrity of renal structures under hypobaric hypoxia. The actins are cytoskeletal proteins that are responsible for cell motility, cell structure and integrity. Besides cell motility, the actin cytoskeleton governs many other cellular activities like cytokinesis, endocytosis and cell adhesion. Similarly, their expression levels were also reported to be affected by ischemia, hypoxia and ATP depletion [57,58]. Taken together, our results strongly indicate that hypobaric hypoxia induced ATP depletion associated with distorted structural cytoskeletal proteins and shift in metabolic pathways together contributed to hypobaric hypoxic kidney injury (Fig 11).

Our results were not able to predict the cause and effect relationship between redox imbalance and energy metabolism. Both of these pathways also linked to each other through oxidative phosphorylation. Nonetheless, it was known that during hypoxia, cells modulate a number of conserved molecular responses and promote altered metabolism to match deficient O_2_ supply in order to maintain cellular homeostasis. Therefore we speculate that redox imbalance and altered energy metabolism may be activating/occurring simultaneously and synergistically contributing to kidney damage.

## Limitations

Limitations of our study are as follows: In our study, the hypobaric hypoxia exposure was limited up to 7 days. Secondly, we have not measured renal hemodynamics and glomerular filtration rate (GFR) in these rats. Third, some of the peptides were not identified by MALDI/LCMS having low Mascot Score that may be due to low concentration of peptides. Fourth, Systolic Blood Pressure by tail cuff method of rats was measured by taking them out of Decompression Chamber at normoxic condition. Fifth, we mainly focused on redox homeostasis under hypobaric hypoxia, thus we have validated only SOD1 and cytoplasmic actin through Western blot and Immunofluroscence. For further confirmation, we have also measured ROS levels, GST activity levels ATP levels and SOD activity levels only in normoxia control and 7 day hypobaric hypoxia group to support our proteomic data. It would have been more appropriate to measure the redox and energetic alterations from the beginning of the study in order to correlate them with the renal structural and biomarkers findings. But we have validated in 7 day hypobaric hypoxia group to confirm our proteomic data. In future, we need to elucidate other mechanisms by validating other proteins under hypoxic damage. Sixth, we need to address therapeutic intervention by blocking redox pathway by ROS scavenger or giving antioxidant to reduce renal damage for future studies. Seventh, although hypobaric hypoxia affects both cortex and medulla region of the kidney, but we have mainly shown renal cortex region in histopathahological and immunohistochemical data.

## Conclusion

Our results demonstrate that sustained hypobaric hypoxia causes the alteration of kidney function with significant structural damage. Sustained hypobaric hypoxia may have ensued early apoptosis, the compensatory over-expression of HIF-1α in renal tissue incited inflammatory and profibrotic pathways. Our proteomic data revealed that the altered regulation of some key proteins responsible for maintaining the cellular functions such as redox homeostasis, cellular integrity and energy metabolism which provides valid explanation for the pathophysiology of hypobaric hypoxia induced kidney damage.

## Supporting information

S1 Fig Effect of hypobaric hypoxia on rat body weight. Changes in body weight in 1, 3 and 7 day of hypobaric hypoxia exposure. Data represented here is Mean ± S. E. M. Values are significant if *P*< 0.05. *stands for level of significance when *P*<0.05, **when *P*<0.05, *** when *P*<0.05 vs. control.
(TIF)

S1 File Result of a Mascot MS/MS search showing query peptide sequences and target hits. (PDF)

## Abbreviations

2DE: Second Dimension Electrophoresis
HH: Hypobaric Hypoxia
MALDI-TOF: Matrix associated laser absorption deionization time of flight
ROS: Reactive Oxygen Species
DCFHDA,2,7: Dichlorofluroscein-Diacetate
MS/MS: Tandem Mass Spectrometry
LC-MS: Liquid Chromatography Mass Spectrometry
GST: Glutathione S Transferase
TUNEL: Terminal Deoxynucleotidyl Transferase dUTPnick end labelling
ATP: Adenosine Triphosphate
DAPI,4,6: Diamidino-2-phenylindole dihydrochloride
KIM-1: Kidney injury molecule -1
SOD-1: Superoxide dismutase 1
ELISA: Enzyme linked Immunosorbant Sandwich assay
GO: Gene Ontology
