# Supplementary figures and images for: Hypobaric hypoxia induced renal damage is mediated by altering redox pathway
Source: PLoS One. 2018 Jul 13;13(7):e0195701. doi: 10.1371/journal.pone.0195701 (PMC6044529; doi:10.1371/journal.pone.0195701)

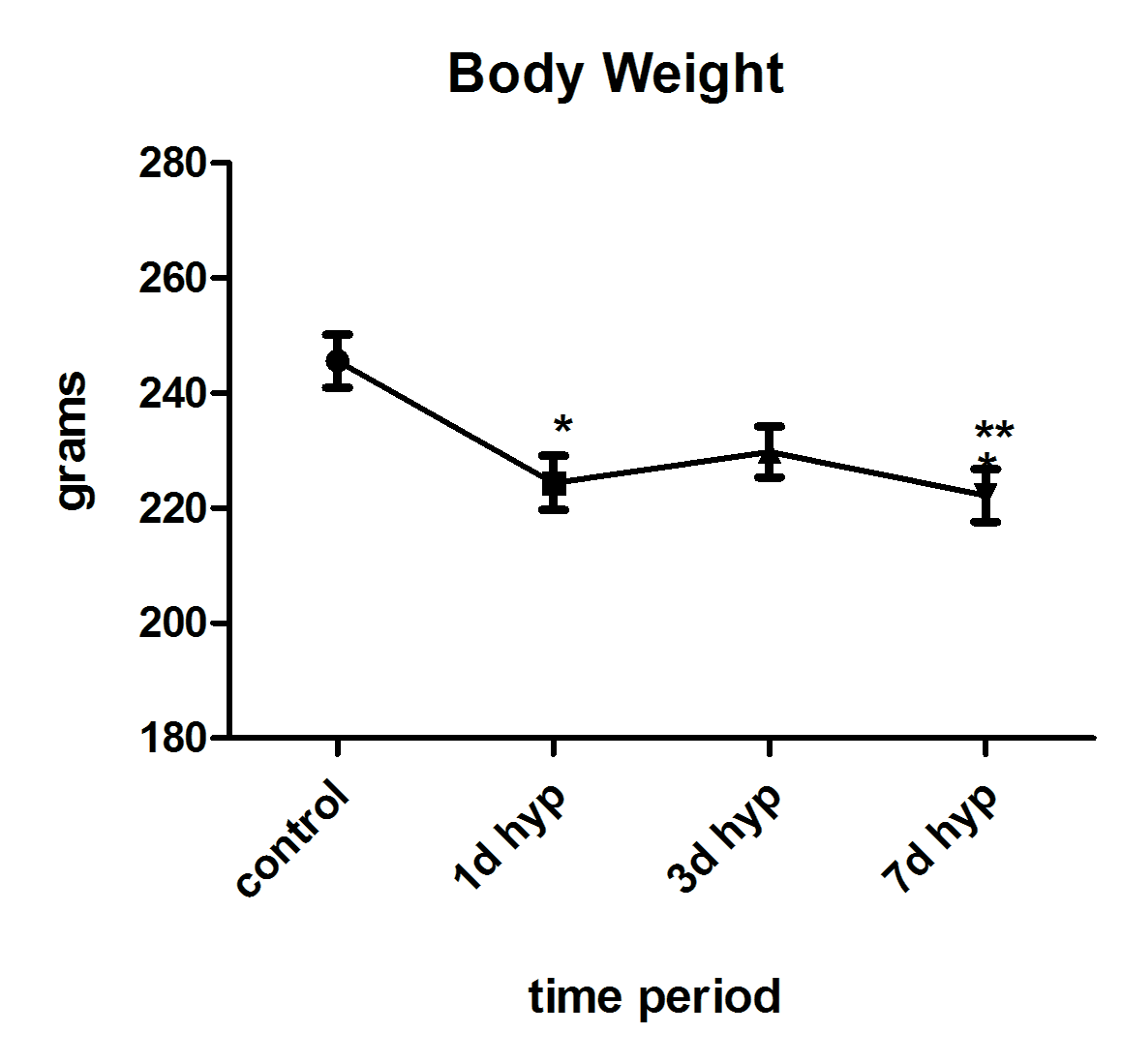

Supplement: S1 Fig — Changes in body weight in 1, 3 and 7 day of hypobaric hypoxia exposure. Data represented here is Mean ± S. E. M. Values are significant if P< 0.05. *stands for level of significance when P<0.05, **when P<0.05, *** when P<0.05 vs. control. (TIF) [file pone.0195701.s001.tif]
