# Supplementary material for: Hypobaric hypoxia induced renal damage is mediated by altering redox pathway
Source: PLoS One. 2018 Jul 13;13(7):e0195701. doi: 10.1371/journal.pone.0195701 (PMC6044529; doi:10.1371/journal.pone.0195701)
Supplement: S1 File — (PDF) [file pone.0195701.s002.pdf]

Mascot Search Results

User	:	
Email	:	
Search title	:	ABSCIEX SERVICE\150916-DRDO\MSMS 2\G4
Database	:	NCBInr 29112011 (13841106 sequences; 4750259772 residues)
Taxonomy	:	Rattus (67132 sequences)
Timestamp	:	22 Sep 2016 at 06:50:35 GMT
Warning	:	A Peptide summary report will usually give a much clearer picture of MS/MS search re
Top Score	:	189 for gi|13591894, alcohol dehydrogenase [NADP+] [Rattus norvegicus]

Probability Based Mowse Score

Protein score is -10*Log(P), where P is the probability that the observed match is a random event.
Protein scores greater than 61 are significant (p<0.05).
Protein scores are derived from ions scores as a non-probabilistic basis for ranking protein hits.

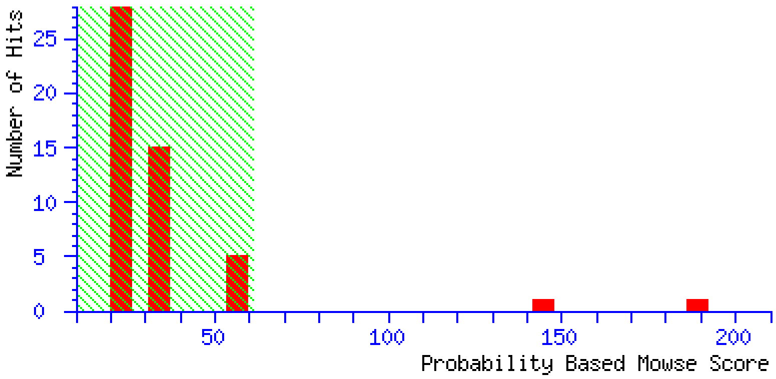

Protein Summary Report

Format As | Protein Summary (deprecated) | Help

Significance threshold p< 0.05 | Max. number of hits 20

Standard scoring ◉ MudPIT scoring ○ | Ions score or expect cut-off 0 | Show sub-sets 0

Show pop-ups ◉ Suppress pop-ups ○ | Sort unassigned Decreasing Score | Require bold red ☐

Re-Search All | Search Unmatched

Index

	Accession	Mass	Score	Description
1.	gi|13591894	36711	189	alcohol dehydrogenase [NADP+] [Rattus norvegicus]
2.	gi|149035585	33369	141	aldo-keto reductase family 1, member A1, isoform CRA_b [Rattus norveg
3.	gi|149031970	21249	61	rCG50690 [Rattus norvegicus]
4.	gi|155369696	59555	60	keratin, type II cytoskeletal 6A [Rattus norvegicus]
5.	gi|293348969	59675	59	PREDICTED: keratin 6A-like [Rattus norvegicus]
6.	gi|554464	3106	58	keratin K6 [Rattus norvegicus]
7.	gi|109482941	59735	55	PREDICTED: keratin, type II cytoskeletal 6A-like isoform 1 [Rattus no

8. **gi|149020956**	22738	36	aldo-keto reductase family 1, member E1, isoform CRA_c [Rattus norveg
9. **gi|149035434**	18109	36	rCG50190, isoform CRA_a [Rattus norvegicus]
10. **gi|149035435**	57721	36	rCG50190, isoform CRA_b [Rattus norvegicus]
11. **gi|149020954**	10429	35	aldo-keto reductase family 1, member E1, isoform CRA_a [Rattus norveg
12. **gi|149041538**	74713	33	rCG58022, isoform CRA_c [Rattus norvegicus]
13. **gi|68341941**	61116	33	importin subunit alpha-6 [Rattus norvegicus]
14. **gi|293362285**	26053	32	PREDICTED: rCG56785-like [Rattus norvegicus]
15. **gi|157823867**	116793	32	tolloid-like 1 [Rattus norvegicus]
16. **gi|149041539**	58151	32	rCG58022, isoform CRA_d [Rattus norvegicus]
17. **gi|149058956**	11702	32	rCG44583 [Rattus norvegicus]
18. **gi|32492560**	106118	31	Aa1017 [Rattus norvegicus]
19. **gi|157821627**	65548	30	SLAIN motif-containing protein 2 [Rattus norvegicus]
20. **gi|149041536**	34338	30	rCG58022, isoform CRA_a [Rattus norvegicus]

Results List

1. gi|13591894 **Mass:** 36711 **Score:** 189 **Expect:** 8.5e-015 **Queries matched:** 19

alcohol dehydrogenase [NADP+] [Rattus norvegicus]

Observed	Mr(expt)	Mr(calc)	ppm	Start	End	Miss	Ions	Peptide
897.6122	896.6049	896.5443	67.6	244 -	251	0	29	R.SPAQILLR.W
897.6122	896.6049	896.5443	67.6	244 -	251	0	---	R.SPAQILLR.W
928.5591	927.5518	927.4814	75.9	35 -	42	0	33	K.YALSVGYR.H
928.5591	927.5518	927.4814	75.9	35 -	42	0	---	K.YALSVGYR.H
1051.6309	1050.6236	1050.5458	74.0	158 -	167	0	---	K.ALGLSNFSSR.Q
1051.6309	1050.6236	1050.5458	74.1	158 -	167	0	---	K.ALGLSNFSSR.Q
1259.7749	1258.7676	1258.6306	109	288 -	297	1	---	K.QLDALNKNWR.Y + 2 Deamidated (NQ)
1259.7749	1258.7676	1258.6306	109	288 -	297	1	---	K.QLDALNKNWR.Y + 2 Deamidated (NQ)
1285.7260	1284.7187	1284.6211	76.0	86 -	96	0	54	K.HHPEDVEPAVR.K
1285.7260	1284.7187	1284.6211	76.0	86 -	96	0	---	K.HHPEDVEPAVR.K
1375.8502	1374.8429	1374.7507	67.1	69 -	80	1	---	K.AVPREELFVTSK.L
1375.8502	1374.8429	1374.7507	67.1	69 -	80	1	---	K.AVPREELFVTSK.L
1413.8215	1412.8142	1412.7160	69.5	86 -	97	1	---	K.HHPEDVEPAVRK.T
1505.8356	1504.8283	1504.6623	110	313 -	325	0	29	R.DAGHPLYPFNDPY.-
1505.8356	1504.8283	1504.6623	110	313 -	325	0	---	R.DAGHPLYPFNDPY.-
1551.8925	1550.8852	1550.7576	82.2	204 -	218	0	---	R.GLEVTAYSPLGSSDR.A
1551.8925	1550.8852	1550.7576	82.3	204 -	218	0	---	R.GLEVTAYSPLGSSDR.A
1707.9005	1706.8932	1706.8814	6.92	295 -	308	1	---	K.NWRYIVPMITVDGK.R + Oxidation (M)
1707.9005	1706.8932	1706.8814	6.93	295 -	308	1	---	K.NWRYIVPMITVDGK.R + Oxidation (M)

No match to: 842.5737, 842.5737, 856.5821, 856.5821, 868.5806, 870.6026, 870.6026, 877.1173, 877.1173, 896.5347, 1179.6890, 1179.6890, 1297.7300, 1308.7545, 1308.7545, 1320.7034, 1323.7662, 1323.7662, 1331.8176, 1331.8176, 1374.7856, 1419.8638, 1419.8638, 1434.8639, 1434.8639, 1463.8888, 1463.8888, 1493.8533, 1493.8533, 1638.9702, 1638.9702, 1791.8708, 1791.8709, 1994.1136, 2050.1382, 2384.1306, 2399.1768, 2705.3350, 2717.2629

2. gi|149035585 **Mass:** 33369 **Score:** 141 **Expect:** 5.3e-010 **Queries matched:** 13

aldo-keto reductase family 1, member A1, isoform CRA_b [Rattus norvegicus]

Observed	Mr(expt)	Mr(calc)	ppm	Start	End	Miss	Ions	Peptide
897.6122	896.6049	896.5443	67.6	244 -	251	0	29	R.SPAQILLR.W
897.6122	896.6049	896.5443	67.6	244 -	251	0	---	R.SPAQILLR.W
928.5591	927.5518	927.4814	75.9	35 -	42	0	33	K.YALSVGYR.H
928.5591	927.5518	927.4814	75.9	35 -	42	0	---	K.YALSVGYR.H
1051.6309	1050.6236	1050.5458	74.0	158 -	167	0	---	K.ALGLSNFSSR.Q
1051.6309	1050.6236	1050.5458	74.1	158 -	167	0	---	K.ALGLSNFSSR.Q

1285.7260	1284.7187	1284.6211	76.0	86 -	96	0	54	K.HHPEDVEPAVR.K
1285.7260	1284.7187	1284.6211	76.0	86 -	96	0	---	K.HHPEDVEPAVR.K
1375.8502	1374.8429	1374.7507	67.1	69 -	80	1	---	K.AVPREELFVTSK.L
1375.8502	1374.8429	1374.7507	67.1	69 -	80	1	---	K.AVPREELFVTSK.L
1413.8215	1412.8142	1412.7160	69.5	86 -	97	1	---	K.HHPEDVEPAVRK.T
1551.8925	1550.8852	1550.7576	82.2	204 -	218	0	---	R.GLEVTAYSPLGSSDR.A
1551.8925	1550.8852	1550.7576	82.3	204 -	218	0	---	R.GLEVTAYSPLGSSDR.A

No match to: 842.5737, 842.5737, 856.5821, 856.5821, 868.5806, 870.6026, 870.6026, 877.1173, 877.1173, 896.5347, 1179.6890, 1179.6890, 1259.7749, 1259.7749, 1297.7300, 1308.7545, 1308.7545, 1320.7034, 1323.7662, 1323.7662, 1331.8176, 1331.8176, 1374.7856, 1419.8638, 1419.8638, 1434.8639, 1434.8639, 1463.8888, 1463.8888, 1493.8533, 1493.8533, 1505.8356, 1505.8356, 1638.9702, 1638.9702, 1707.9005, 1707.9005, 1791.8708, 1791.8709, 1994.1136, 2050.1382, 2384.1306, 2399.1768, 2705.3350, 2717.2629

3. gi|149031970 **Mass:** 21249 **Score:** 61 **Expect:** 0.058 **Queries matched:** 6
rCG50690 [Rattus norvegicus]

Observed	Mr(expt)	Mr(calc)	ppm	Start	End	Miss	Ions	Peptide
1179.6890	1178.6817	1178.5931	75.1	119 -	128	0	51	K.YEELQITAGR.H
1179.6890	1178.6817	1178.5931	75.2	119 -	128	0	---	K.YEELQITAGR.H
1285.7260	1284.7187	1284.6020	90.8	1 -	11	1	---	-.MQGTVEDYKSK.Y
1285.7260	1284.7187	1284.6020	90.9	1 -	11	1	---	-.MQGTVEDYKSK.Y
1331.8176	1330.8103	1330.6841	94.9	135 -	145	1	---	R.NTKQEISEINR.M
1331.8176	1330.8104	1330.6841	94.9	135 -	145	1	---	R.NTKQEISEINR.M

No match to: 842.5737, 842.5737, 856.5821, 856.5821, 868.5806, 870.6026, 870.6026, 877.1173, 877.1173, 896.5347, 897.6122, 897.6122, 928.5591, 928.5591, 1051.6309, 1051.6309, 1259.7749, 1259.7749, 1297.7300, 1308.7545, 1308.7545, 1320.7034, 1323.7662, 1323.7662, 1374.7856, 1375.8502, 1375.8502, 1413.8215, 1419.8638, 1419.8638, 1434.8639, 1434.8639, 1463.8888, 1463.8888, 1493.8533, 1493.8533, 1505.8356, 1505.8356, 1551.8925, 1551.8925, 1638.9702, 1638.9702, 1707.9005, 1707.9005, 1791.8708, 1791.8709, 1994.1136, 2050.1382, 2384.1306, 2399.1768, 2705.3350, 2717.2629

4. gi|155369696 **Mass:** 59555 **Score:** 60 **Expect:** 0.063 **Queries matched:** 11
keratin, type II cytoskeletal 6A [Rattus norvegicus]

Observed	Mr(expt)	Mr(calc)	ppm	Start	End	Miss	Ions	Peptide
1051.6309	1050.6236	1050.4764	140	58 -	67	0	---	R.SLCGVGSSQR.I + Deamidated (NQ)
1051.6309	1050.6236	1050.4764	140	58 -	67	0	---	R.SLCGVGSSQR.I + Deamidated (NQ)
1179.6890	1178.6817	1178.5931	75.1	349 -	358	0	51	K.YEELQITAGR.H
1179.6890	1178.6817	1178.5931	75.2	349 -	358	0	---	K.YEELQITAGR.H
1331.8176	1330.8103	1330.6841	94.9	365 -	375	1	---	R.NTKQEISEINR.M
1331.8176	1330.8104	1330.6841	94.9	365 -	375	1	---	R.NTKQEISEINR.M
1463.8888	1462.8815	1462.7667	78.5	172 -	183	1	---	R.FLEQQNKVLDTK.W + Deamidated (NQ
1463.8888	1462.8815	1462.7667	78.5	172 -	183	1	---	R.FLEQQNKVLDTK.W + Deamidated (NQ
1493.8533	1492.8460	1492.7959	33.5	433 -	444	1	---	R.LLKEYQDLMNVK.L
1493.8533	1492.8460	1492.7959	33.6	433 -	444	1	---	R.LLKEYQDLMNVK.L
2384.1306	2383.1233	2383.2093	-36.08	436 -	455	1	---	K.EYQDLMNVKLALDVEIATYR.T

No match to: 842.5737, 842.5737, 856.5821, 856.5821, 868.5806, 870.6026, 870.6026, 877.1173, 877.1173, 896.5347, 897.6122, 897.6122, 928.5591, 928.5591, 1259.7749, 1259.7749, 1285.7260, 1285.7260, 1297.7300, 1308.7545, 1308.7545, 1320.7034, 1323.7662, 1323.7662, 1374.7856, 1375.8502, 1375.8502, 1413.8215, 1419.8638, 1419.8638, 1434.8639, 1434.8639, 1505.8356, 1505.8356, 1551.8925, 1551.8925, 1638.9702, 1638.9702, 1707.9005, 1707.9005, 1791.8708, 1791.8709, 1994.1136, 2050.1382, 2399.1768, 2705.3350, 2717.2629

5. gi|293348969 **Mass:** 59675 **Score:** 59 **Expect:** 0.088 **Queries matched:** 10
PREDICTED: keratin 6A-like [Rattus norvegicus]

Observed	Mr(expt)	Mr(calc)	ppm	Start		End	Miss	Ions	Peptide
1179.6890	1178.6817	1178.5931	75.1	349	-	358	0	51	K.YEELQITAGR.H
1179.6890	1178.6817	1178.5931	75.2	349	-	358	0	---	K.YEELQITAGR.H
1331.8176	1330.8103	1330.6841	94.9	365	-	375	1	---	R.NTKQEISEINR.M
1331.8176	1330.8104	1330.6841	94.9	365	-	375	1	---	R.NTKQEISEINR.M
1463.8888	1462.8815	1462.7667	78.5	172	-	183	1	---	R.FLEQQNKVLDTK.W + Deamidated (NQ
1463.8888	1462.8815	1462.7667	78.5	172	-	183	1	---	R.FLEQQNKVLDTK.W + Deamidated (NQ
1493.8533	1492.8460	1492.7959	33.5	433	-	444	1	---	R.LLKEYQDLMNVK.L
1493.8533	1492.8460	1492.7959	33.6	433	-	444	1	---	R.LLKEYQDLMNVK.L
2050.1382	2049.1309	2049.0166	55.8	197	-	212	1	---	R.QNLEPLFEQYINNLRR.Q + 3 Deamidat
2384.1306	2383.1233	2383.2093	-36.08	436	-	455	1	---	K.EYQDLMNVKLALDVEIATYR.T

No match to: 842.5737, 842.5737, 856.5821, 856.5821, 868.5806, 870.6026, 870.6026, 877.1173, 877.1173, 896.5347, 897.6122, 897.6122, 928.5591, 928.5591, 1051.6309, 1051.6309, 1259.7749, 1259.7749, 1285.7260, 1285.7260, 1297.7300, 1308.7545, 1308.7545, 1320.7034, 1323.7662, 1323.7662, 1374.7856, 1375.8502, 1375.8502, 1413.8215, 1419.8638, 1419.8638, 1434.8639, 1434.8639, 1505.8356, 1505.8356, 1551.8925, 1551.8925, 1638.9702, 1638.9702, 1707.9005, 1707.9005, 1791.8708, 1791.8709, 1994.1136, 2399.1768, 2705.3350, 2717.2629

6. gi|554464 **Mass:** 3106 **Score:** 58 **Expect:** 0.11 **Queries matched:** 2
keratin K6 [Rattus norvegicus]

Observed	Mr(expt)	Mr(calc)	ppm	Start		End	Miss	Ions	Peptide
1179.6890	1178.6817	1178.5931	75.1	5	-	14	0	51	K.YEELQITAGR.H
1179.6890	1178.6817	1178.5931	75.2	5	-	14	0	---	K.YEELQITAGR.H

No match to: 842.5737, 842.5737, 856.5821, 856.5821, 868.5806, 870.6026, 870.6026, 877.1173, 877.1173, 896.5347, 897.6122, 897.6122, 928.5591, 928.5591, 1051.6309, 1051.6309, 1259.7749, 1259.7749, 1285.7260, 1285.7260, 1297.7300, 1308.7545, 1308.7545, 1320.7034, 1323.7662, 1323.7662, 1331.8176, 1331.8176, 1374.7856, 1375.8502, 1375.8502, 1413.8215, 1419.8638, 1419.8638, 1434.8639, 1434.8639, 1463.8888, 1463.8888, 1493.8533, 1493.8533, 1505.8356, 1505.8356, 1551.8925, 1551.8925, 1638.9702, 1638.9702, 1707.9005, 1707.9005, 1791.8708, 1791.8709, 1994.1136, 2050.1382, 2384.1306, 2399.1768, 2705.3350, 2717.2629

7. gi|109482941 **Mass:** 59735 **Score:** 55 **Expect:** 0.2 **Queries matched:** 12
PREDICTED: keratin, type II cytoskeletal 6A-like isoform 1 [Rattus norvegicus]

Observed	Mr(expt)	Mr(calc)	ppm	Start		End	Miss	Ions	Peptide
1179.6890	1178.6817	1178.5931	75.1	349	-	358	0	51	K.YEELQITAGR.H
1179.6890	1178.6817	1178.5931	75.2	349	-	358	0	---	K.YEELQITAGR.H
1331.8176	1330.8103	1330.6841	94.9	365	-	375	1	---	R.NTKQEISEINR.M
1331.8176	1330.8104	1330.6841	94.9	365	-	375	1	---	R.NTKQEISEINR.M
1463.8888	1462.8815	1462.7667	78.5	172	-	183	1	---	R.FLEQQNKVLDTK.W + Deamidated (NQ
1463.8888	1462.8815	1462.7667	78.5	172	-	183	1	---	R.FLEQQNKVLDTK.W + Deamidated (NQ
1493.8533	1492.8460	1492.7959	33.5	433	-	444	1	---	R.LLKEYQDLMNVK.L
1493.8533	1492.8460	1492.7959	33.6	433	-	444	1	---	R.LLKEYQDLMNVK.L
1551.8925	1550.8852	1550.7763	70.2	289	-	301	0	---	R.VIFEAELSQMQTR.I
1551.8925	1550.8852	1550.7763	70.3	289	-	301	0	---	R.VIFEAELSQMQTR.I
1994.1136	1993.1063	1993.0381	34.2	197	-	212	1	---	R.QGLETLFEQYINNLRR.Q
2384.1306	2383.1233	2383.2093	-36.08	436	-	455	1	---	K.EYQDLMNVKLALDVEIATYR.T

No match to: 842.5737, 842.5737, 856.5821, 856.5821, 868.5806, 870.6026, 870.6026, 877.1173, 877.1173, 896.5347, 897.6122, 897.6122, 928.5591, 928.5591, 1051.6309, 1051.6309, 1259.7749, 1259.7749, 1285.7260, 1285.7260, 1297.7300, 1308.7545, 1308.7545, 1320.7034, 1323.7662, 1323.7662, 1374.7856, 1375.8502, 1375.8502, 1413.8215, 1419.8638, 1419.8638, 1434.8639, 1434.8639, 1505.8356, 1505.8356, 1638.9702, 1638.9702, 1707.9005, 1707.9005, 1791.8708, 1791.8709, 2050.1382, 2399.1768, 2705.3350, 2717.2629

8. gi|149020956 **Mass:** 22738 **Score:** 36 **Expect:** 15 **Queries matched:** 4
aldo-keto reductase family 1, member E1, isoform CRA_c [Rattus norvegicus]

Observed	Mr(expt)	Mr(calc)	ppm	Start	End	Miss	Ions	Peptide
897.6122	896.6049	896.5443	67.6	125 -	132	0	29	K.SPAQILIR.F
897.6122	896.6049	896.5443	67.6	125 -	132	0	---	K.SPAQILIR.F
928.5591	927.5518	927.5250	28.9	145 -	152	1	---	K.SVNPSRIR.E
928.5591	927.5518	927.5250	28.9	145 -	152	1	---	K.SVNPSRIR.E

No match to: 842.5737, 842.5737, 856.5821, 856.5821, 868.5806, 870.6026, 870.6026, 877.1173, 877.1173, 896.5347, 1051.6309, 1051.6309, 1179.6890, 1179.6890, 1259.7749, 1259.7749, 1285.7260, 1285.7260, 1297.7300, 1308.7545, 1308.7545, 1320.7034, 1323.7662, 1323.7662, 1331.8176, 1331.8176, 1374.7856, 1375.8502, 1375.8502, 1413.8215, 1419.8638, 1419.8638, 1434.8639, 1434.8639, 1463.8888, 1463.8888, 1493.8533, 1493.8533, 1505.8356, 1505.8356, 1551.8925, 1551.8925, 1638.9702, 1638.9702, 1707.9005, 1707.9005, 1791.8708, 1791.8709, 1994.1136, 2050.1382, 2384.1306, 2399.1768, 2705.3350, 2717.2629

9. gi|149035434 **Mass:** 18109 **Score:** 36 **Expect:** 18 **Queries matched:** 6
rCG50190, isoform CRA_a [Rattus norvegicus]

Observed	Mr(expt)	Mr(calc)	ppm	Start	End	Miss	Ions	Peptide
842.5737	841.5664	841.4657	120	71 -	77	0	---	K.EIQSLPR.A
842.5737	841.5665	841.4657	120	71 -	77	0	26	K.EIQSLPR.A
1259.7749	1258.7676	1258.6768	72.1	94 -	104	1	---	R.ENSELEAKVLK.F
1259.7749	1258.7676	1258.6768	72.1	94 -	104	1	---	R.ENSELEAKVLK.F
1493.8533	1492.8460	1492.7885	38.5	37 -	49	1	---	K.RQQLTSAFTAIQK.S + 2 Deamidated (
1493.8533	1492.8460	1492.8038	28.3	82 -	93	0	---	R.EQFLVAYNLLQR.E

No match to: 856.5821, 856.5821, 868.5806, 870.6026, 870.6026, 877.1173, 877.1173, 896.5347, 897.6122, 897.6122, 928.5591, 928.5591, 1051.6309, 1051.6309, 1179.6890, 1179.6890, 1285.7260, 1285.7260, 1297.7300, 1308.7545, 1308.7545, 1320.7034, 1323.7662, 1323.7662, 1331.8176, 1331.8176, 1374.7856, 1375.8502, 1375.8502, 1413.8215, 1419.8638, 1419.8638, 1434.8639, 1434.8639, 1463.8888, 1463.8888, 1505.8356, 1505.8356, 1551.8925, 1551.8925, 1638.9702, 1638.9702, 1707.9005, 1707.9005, 1791.8708, 1791.8709, 1994.1136, 2050.1382, 2384.1306, 2399.1768, 2705.3350, 2717.2629

10. gi|149035435 **Mass:** 57721 **Score:** 36 **Expect:** 18 **Queries matched:** 8
rCG50190, isoform CRA_b [Rattus norvegicus]

Observed	Mr(expt)	Mr(calc)	ppm	Start	End	Miss	Ions	Peptide
842.5737	841.5664	841.4657	120	71 -	77	0	---	K.EIQSLPR.A
842.5737	841.5665	841.4657	120	71 -	77	0	26	K.EIQSLPR.A
1259.7749	1258.7676	1258.6768	72.1	94 -	104	1	---	R.ENSELEAKVLK.F
1259.7749	1258.7676	1258.6768	72.1	94 -	104	1	---	R.ENSELEAKVLK.F
1285.7260	1284.7187	1284.6786	31.2	292 -	302	1	---	K.QAQEKLLDNAR.M
1285.7260	1284.7187	1284.6786	31.3	292 -	302	1	---	K.QAQEKLLDNAR.M
1493.8533	1492.8460	1492.7885	38.5	37 -	49	1	---	K.RQQLTSAFTAIQK.S + 2 Deamidated (
1493.8533	1492.8460	1492.8038	28.3	82 -	93	0	---	R.EQFLVAYNLLQR.E

No match to: 856.5821, 856.5821, 868.5806, 870.6026, 870.6026, 877.1173, 877.1173, 896.5347, 897.6122, 897.6122, 928.5591, 928.5591, 1051.6309, 1051.6309, 1179.6890, 1179.6890, 1297.7300, 1308.7545, 1308.7545, 1320.7034, 1323.7662, 1323.7662, 1331.8176, 1331.8176, 1374.7856, 1375.8502, 1375.8502, 1413.8215, 1419.8638, 1419.8638, 1434.8639, 1434.8639, 1463.8888, 1463.8888, 1505.8356, 1505.8356, 1551.8925, 1551.8925, 1638.9702, 1638.9702, 1707.9005, 1707.9005, 1791.8708, 1791.8709, 1994.1136, 2050.1382, 2384.1306, 2399.1768, 2705.3350, 2717.2629

11. gi|149020954 **Mass:** 10429 **Score:** 35 **Expect:** 21 **Queries matched:** 4
aldo-keto reductase family 1, member E1, isoform CRA_a [Rattus norvegicus]

Observed	Mr(expt)	Mr(calc)	ppm	Start	End	Miss	Ions	Peptide

897.6122	896.6049	896.5443	67.6	16 - 23	0	29	K.SPAQILIR.F
897.6122	896.6049	896.5443	67.6	16 - 23	0	---	K.SPAQILIR.F
928.5591	927.5518	927.5250	28.9	36 - 43	1	---	K.SVNPSRIR.E
928.5591	927.5518	927.5250	28.9	36 - 43	1	---	K.SVNPSRIR.E

No match to: 842.5737, 842.5737, 856.5821, 856.5821, 868.5806, 870.6026, 870.6026, 877.1173, 877.1173, 896.5347, 1051.6309, 1051.6309, 1179.6890, 1179.6890, 1259.7749, 1259.7749, 1285.7260, 1285.7260, 1297.7300, 1308.7545, 1308.7545, 1320.7034, 1323.7662, 1323.7662, 1331.8176, 1331.8176, 1374.7856, 1375.8502, 1375.8502, 1413.8215, 1419.8638, 1419.8638, 1434.8639, 1434.8639, 1463.8888, 1463.8888, 1493.8533, 1493.8533, 1505.8356, 1505.8356, 1551.8925, 1551.8925, 1638.9702, 1638.9702, 1707.9005, 1707.9005, 1791.8708, 1791.8709, 1994.1136, 2050.1382, 2384.1306, 2399.1768, 2705.3350, 2717.2629

12. gi|149041538 **Mass:** 74713 **Score:** 33 **Expect:** 36 **Queries matched:** 10

rCG58022, isoform CRA_c [Rattus norvegicus]

Observed	Mr(expt)	Mr(calc)	ppm	Start - End	Miss	Ions	Peptide
842.5737	841.5664	841.5637	3.28	360 - 366	1	---	R.KELLVLK.L
842.5737	841.5665	841.5637	3.32	360 - 366	1	---	R.KELLVLK.L
1179.6890	1178.6817	1178.5931	75.1	240 - 248	1	24	R.YQEVLDKQR.Q + Deamidated (NQ)
1179.6890	1178.6817	1178.5931	75.2	240 - 248	1	---	R.YQEVLDKQR.Q + Deamidated (NQ)
1323.7662	1322.7589	1322.5772	137	143 - 153	1	---	K.QLASRNCSEEK.C + 2 Deamidated (NQ
1323.7662	1322.7590	1322.5772	137	143 - 153	1	---	K.QLASRNCSEEK.C + 2 Deamidated (NQ
1413.8215	1412.8142	1412.8126	1.16	361 - 372	1	---	K.ELLVLKLEEAEK.E
1463.8888	1462.8815	1462.7126	115	321 - 331	1	---	R.EVWEMELDRLK.N + Oxidation (M)
1463.8888	1462.8815	1462.7126	115	321 - 331	1	---	R.EVWEMELDRLK.N + Oxidation (M)
1994.1136	1993.1063	1992.8946	106	46 - 63	0	---	K.NLSPPSVSSQMITEESNR.N + 2 Deamida

No match to: 856.5821, 856.5821, 868.5806, 870.6026, 870.6026, 877.1173, 877.1173, 896.5347, 897.6122, 897.6122, 928.5591, 928.5591, 1051.6309, 1051.6309, 1259.7749, 1259.7749, 1285.7260, 1285.7260, 1297.7300, 1308.7545, 1308.7545, 1320.7034, 1331.8176, 1331.8176, 1374.7856, 1375.8502, 1375.8502, 1419.8638, 1419.8638, 1434.8639, 1434.8639, 1493.8533, 1493.8533, 1505.8356, 1505.8356, 1551.8925, 1551.8925, 1638.9702, 1638.9702, 1707.9005, 1707.9005, 1791.8708, 1791.8709, 2050.1382, 2384.1306, 2399.1768, 2705.3350, 2717.2629

13. gi|68341941 **Mass:** 61116 **Score:** 33 **Expect:** 37 **Queries matched:** 8

importin subunit alpha-6 [Rattus norvegicus]

Observed	Mr(expt)	Mr(calc)	ppm	Start - End	Miss	Ions	Peptide
842.5737	841.5664	841.4770	106	48 - 54	1	---	R.RNVSLPR.N + Deamidated (NQ)
842.5737	841.5665	841.4770	106	48 - 54	1	34	R.RNVSLPR.N + Deamidated (NQ)
1374.7856	1373.7783	1373.7013	56.1	299 - 309	0	---	R.LVELLMHNDYK.V
1375.8502	1374.8429	1374.6853	115	299 - 309	0	---	R.LVELLMHNDYK.V + Deamidated (NQ)
1375.8502	1374.8429	1374.6853	115	299 - 309	0	---	R.LVELLMHNDYK.V + Deamidated (NQ)
1413.8215	1412.8142	1412.8391	-17.64	162 - 174	0	---	K.VVIETGAVPIFIR.L
1551.8925	1550.8852	1550.9144	-18.84	437 - 450	0	---	K.IVQVALNGLENILR.L
1551.8925	1550.8852	1550.9144	-18.81	437 - 450	0	---	K.IVQVALNGLENILR.L

No match to: 856.5821, 856.5821, 868.5806, 870.6026, 870.6026, 877.1173, 877.1173, 896.5347, 897.6122, 897.6122, 928.5591, 928.5591, 1051.6309, 1051.6309, 1179.6890, 1179.6890, 1259.7749, 1259.7749, 1285.7260, 1285.7260, 1297.7300, 1308.7545, 1308.7545, 1320.7034, 1323.7662, 1323.7662, 1331.8176, 1331.8176, 1419.8638, 1419.8638, 1434.8639, 1434.8639, 1463.8888, 1463.8888, 1493.8533, 1493.8533, 1505.8356, 1505.8356, 1638.9702, 1638.9702, 1707.9005, 1707.9005, 1791.8708, 1791.8709, 1994.1136, 2050.1382, 2384.1306, 2399.1768, 2705.3350, 2717.2629

14. gi|293362285 **Mass:** 26053 **Score:** 32 **Expect:** 40 **Queries matched:** 7

PREDICTED: rCG56785-like [Rattus norvegicus]

Observed	Mr(expt)	Mr(calc)	ppm	Start	End	Miss	Ions	Peptide

1259.7749	1258.7676	1258.6517	92.1	73 -	82	1	---	K.LITEERNDLR.D + Deamidated (NQ)
1259.7749	1258.7676	1258.6517	92.1	73 -	82	1	---	K.LITEERNDLR.D + Deamidated (NQ)
1308.7545	1307.7472	1307.6081	106	36 -	46	0	---	R.QQFWGMLNAGR.E + Deamidated (NQ)
1308.7545	1307.7472	1307.6081	106	36 -	46	0	25	R.QQFWGMLNAGR.E + Deamidated (NQ)
1323.7662	1322.7589	1322.6190	106	36 -	46	0	---	R.QQFWGMLNAGR.E + Oxidation (M)
1323.7662	1322.7590	1322.6190	106	36 -	46	0	---	R.QQFWGMLNAGR.E + Oxidation (M)
2705.3350	2704.3277	2704.1194	77.0	23 -	46	1	---	K.ESSLSSQSNDGQRQQFWGMLNAGR.E + 6 D

No match to: 842.5737, 842.5737, 856.5821, 856.5821, 868.5806, 870.6026, 870.6026, 877.1173, 877.1173, 896.5347, 897.6122, 897.6122, 928.5591, 928.5591, 1051.6309, 1051.6309, 1179.6890, 1179.6890, 1285.7260, 1285.7260, 1297.7300, 1320.7034, 1331.8176, 1331.8176, 1374.7856, 1375.8502, 1375.8502, 1413.8215, 1419.8638, 1419.8638, 1434.8639, 1434.8639, 1463.8888, 1463.8888, 1493.8533, 1493.8533, 1505.8356, 1505.8356, 1551.8925, 1551.8925, 1638.9702, 1638.9702, 1707.9005, 1707.9005, 1791.8708, 1791.8709, 1994.1136, 2050.1382, 2384.1306, 2399.1768, 2717.2629

15. gi|157823867 **Mass:** 116793 **Score:** 32 **Expect:** 40 **Queries matched:** 17

tolloid-like 1 [Rattus norvegicus]

Observed	Mr(expt)	Mr(calc)	ppm	Start	End	Miss	Ions	Peptide
842.5737	841.5664	841.4657	120	2 -	9	0	---	M.GLQALSPR.M + Deamidated (NQ)
842.5737	841.5665	841.4657	120	2 -	9	0	21	M.GLQALSPR.M + Deamidated (NQ)
897.6122	896.6049	896.4214	205	439 -	444	0	---	R.MWIEFR.S + Oxidation (M)
897.6122	896.6049	896.4214	205	439 -	444	0	---	R.MWIEFR.S + Oxidation (M)
1259.7749	1258.7676	1258.5789	150	524 -	535	0	---	R.DGNSENSPLIGR.F + Deamidated (NQ
1259.7749	1258.7676	1258.5789	150	524 -	535	0	---	R.DGNSENSPLIGR.F + Deamidated (NQ
1285.7260	1284.7187	1284.6714	36.8	625 -	636	0	---	K.LNGTITTPGWPK.E + Deamidated (NQ
1285.7260	1284.7187	1284.6714	36.8	625 -	636	0	---	K.LNGTITTPGWPK.E + Deamidated (NQ
1419.8638	1418.8565	1418.7001	110	117 -	129	1	---	R.RIGSGLEQNNTTK.G + 2 Deamidated
1419.8638	1418.8565	1418.7001	110	117 -	129	1	---	R.RIGSGLEQNNTTK.G + 2 Deamidated
1434.8639	1433.8566	1433.5551	210	767 -	777	1	---	K.HDCKEAECEQK.I + Deamidated (NQ)
1434.8639	1433.8566	1433.5551	210	767 -	777	1	---	K.HDCKEAECEQK.I + Deamidated (NQ)
1463.8888	1462.8815	1462.6510	158	1002 -	1013	1	---	K.SIRYPETMHAEN.- + Oxidation (M)
1463.8888	1462.8815	1462.6510	158	1002 -	1013	1	---	K.SIRYPETMHAEN.- + Oxidation (M)
2050.1382	2049.1309	2049.0353	46.7	643 -	659	1	---	K.NCVWQVIAPSQYRISVK.F + 2 Deamida
2705.3350	2704.3277	2704.1356	71.0	197 -	218	0	---	R.SDEESYIVFTYRPCGCCSYVGR.R
2717.2629	2716.2556	2716.1897	24.3	279 -	301	1	---	K.MEPGEVNSLGERYDFDSIMHYAR.N + Dea

No match to: 856.5821, 856.5821, 868.5806, 870.6026, 870.6026, 877.1173, 877.1173, 896.5347, 928.5591, 928.5591, 1051.6309, 1051.6309, 1179.6890, 1179.6890, 1297.7300, 1308.7545, 1308.7545, 1320.7034, 1323.7662, 1323.7662, 1331.8176, 1331.8176, 1374.7856, 1375.8502, 1375.8502, 1413.8215, 1493.8533, 1493.8533, 1505.8356, 1505.8356, 1551.8925, 1551.8925, 1638.9702, 1638.9702, 1707.9005, 1707.9005, 1791.8708, 1791.8709, 1994.1136, 2384.1306, 2399.1768

16. gi|149041539 **Mass:** 58151 **Score:** 32 **Expect:** 40 **Queries matched:** 7

rCG58022, isoform CRA_d [Rattus norvegicus]

Observed	Mr(expt)	Mr(calc)	ppm	Start	End	Miss	Ions	Peptide
842.5737	841.5664	841.5637	3.28	205 -	211	1	---	R.KELLVLK.L
842.5737	841.5665	841.5637	3.32	205 -	211	1	---	R.KELLVLK.L
1179.6890	1178.6817	1178.5931	75.1	85 -	93	1	24	R.YQEVLDKQR.Q + Deamidated (NQ)
1179.6890	1178.6817	1178.5931	75.2	85 -	93	1	---	R.YQEVLDKQR.Q + Deamidated (NQ)
1413.8215	1412.8142	1412.8126	1.16	206 -	217	1	---	K.ELLVLKLEEAEK.E
1463.8888	1462.8815	1462.7126	115	166 -	176	1	---	R.EVWEMELDRLK.N + Oxidation (M)
1463.8888	1462.8815	1462.7126	115	166 -	176	1	---	R.EVWEMELDRLK.N + Oxidation (M)

No match to: 856.5821, 856.5821, 868.5806, 870.6026, 870.6026, 877.1173, 877.1173, 896.5347,

897.6122, 897.6122, 928.5591, 928.5591, 1051.6309, 1051.6309, 1259.7749, 1259.7749, 1285.7260, 1285.7260, 1297.7300, 1308.7545, 1308.7545, 1320.7034, 1323.7662, 1323.7662, 1331.8176, 1331.8176, 1374.7856, 1375.8502, 1375.8502, 1419.8638, 1419.8638, 1434.8639, 1434.8639, 1493.8533, 1493.8533, 1505.8356, 1505.8356, 1551.8925, 1551.8925, 1638.9702, 1638.9702, 1707.9005, 1707.9005, 1791.8708, 1791.8709, 1994.1136, 2050.1382, 2384.1306, 2399.1768, 2705.3350, 2717.2629

17. gi|149058956 **Mass:** 11702 **Score:** 32 **Expect:** 43 **Queries matched:** 7
rCG44583 [Rattus norvegicus]

Observed	Mr(expt)	Mr(calc)	ppm	Start	End	Miss	Ions	Peptide
897.6122	896.6049	896.4942	123	1 -	7	0	---	-.MALFFLR.G
897.6122	896.6049	896.4942	123	1 -	7	0	---	-.MALFFLR.G
1179.6890	1178.6817	1178.5567	106	20 -	28	0	---	R.QELNYLENR.K + Deamidated (NQ)
1179.6890	1178.6817	1178.5567	106	20 -	28	0	---	R.QELNYLENR.K + Deamidated (NQ)
1308.7545	1307.7472	1307.6357	85.3	20 -	29	1	---	R.QELNYLENRK.T + 2 Deamidated (NQ)
1308.7545	1307.7472	1307.6357	85.3	20 -	29	1	15	R.QELNYLENRK.T + 2 Deamidated (NQ)
1994.1136	1993.1063	1993.0713	17.6	42 -	56	1	---	K.WDFLKWLLNLFIEEK.I

No match to: 842.5737, 842.5737, 856.5821, 856.5821, 868.5806, 870.6026, 870.6026, 877.1173, 877.1173, 896.5347, 928.5591, 928.5591, 1051.6309, 1051.6309, 1259.7749, 1259.7749, 1285.7260, 1285.7260, 1297.7300, 1320.7034, 1323.7662, 1323.7662, 1331.8176, 1331.8176, 1374.7856, 1375.8502, 1375.8502, 1413.8215, 1419.8638, 1419.8638, 1434.8639, 1434.8639, 1463.8888, 1463.8888, 1493.8533, 1493.8533, 1505.8356, 1505.8356, 1551.8925, 1551.8925, 1638.9702, 1638.9702, 1707.9005, 1707.9005, 1791.8708, 1791.8709, 2050.1382, 2384.1306, 2399.1768, 2705.3350, 2717.2629

18. gi|32492560 **Mass:** 106118 **Score:** 31 **Expect:** 58 **Queries matched:** 16
Aa1017 [Rattus norvegicus]

Observed	Mr(expt)	Mr(calc)	ppm	Start	End	Miss	Ions	Peptide
842.5737	841.5664	841.4844	97.5	427 -	433	0	---	R.ACLLLPR.G
842.5737	841.5665	841.4844	97.5	427 -	433	0	14	R.ACLLLPR.G
856.5821	855.5748	855.4814	109	360 -	366	1	2	R.RNLPQVK.E + 2 Deamidated (NQ)
856.5821	855.5748	855.4814	109	360 -	366	1	---	R.RNLPQVK.E + 2 Deamidated (NQ)
870.6026	869.5953	869.5446	58.3	570 -	576	1	---	K.IILARER.K
870.6026	869.5953	869.5446	58.3	570 -	576	1	---	K.IILARER.K
1297.7300	1296.7227	1296.5510	132	560 -	569	0	---	K.FDNQEDLWTK.I + 2 Deamidated (NQ)
1419.8638	1418.8565	1418.7266	91.6	361 -	372	1	---	R.NLPQVKEDSPHR.E
1419.8638	1418.8565	1418.7266	91.6	361 -	372	1	---	R.NLPQVKEDSPHR.E
1434.8639	1433.8566	1433.7527	72.4	129 -	139	1	---	K.IHHQNIKQYPR.M + Deamidated (NQ)
1434.8639	1433.8566	1433.7527	72.5	129 -	139	1	---	K.IHHQNIKQYPR.M + Deamidated (NQ)
1463.8888	1462.8815	1462.7528	88.0	318 -	331	0	---	R.VEVIPAGSSPHTNR.R
1463.8888	1462.8815	1462.7528	88.0	318 -	331	0	---	R.VEVIPAGSSPHTNR.R
1551.8925	1550.8852	1550.6871	128	282 -	293	0	---	K.HGCCLVTHPEWR.T
1551.8925	1550.8852	1550.6871	128	282 -	293	0	---	K.HGCCLVTHPEWR.T
2050.1382	2049.1309	2048.9916	68.0	196 -	215	1	---	R.GTGNIQLPGFSGGTWGSERK.K + Deamida

No match to: 868.5806, 877.1173, 877.1173, 896.5347, 897.6122, 897.6122, 928.5591, 928.5591, 1051.6309, 1051.6309, 1179.6890, 1179.6890, 1259.7749, 1259.7749, 1285.7260, 1285.7260, 1308.7545, 1308.7545, 1320.7034, 1323.7662, 1323.7662, 1331.8176, 1331.8176, 1374.7856, 1375.8502, 1375.8502, 1413.8215, 1493.8533, 1493.8533, 1505.8356, 1505.8356, 1638.9702, 1638.9702, 1707.9005, 1707.9005, 1791.8708, 1791.8709, 1994.1136, 2384.1306, 2399.1768, 2705.3350, 2717.2629

19. gi|157821627 **Mass:** 65548 **Score:** 30 **Expect:** 72 **Queries matched:** 8
SLAIN motif-containing protein 2 [Rattus norvegicus]

Observed	Mr(expt)	Mr(calc)	ppm	Start	End	Miss	Ions	Peptide

868.5806	867.5733	867.5290	51.1	580	- 586	1	---	K.LVQPVRR.S + Deamidated (NQ)
1179.6890	1178.6817	1178.5965	72.3	174	- 183	1	23	K.LDQTMSALKR.Q + Deamidated (NQ);
1179.6890	1178.6817	1178.5965	72.3	174	- 183	1	---	K.LDQTMSALKR.Q + Deamidated (NQ);
1463.8888	1462.8815	1462.7086	118	448	- 460	1	---	R.MQPQASATSQRLK.S + 2 Deamidated (
1463.8888	1462.8815	1462.7086	118	448	- 460	1	---	R.MQPQASATSQRLK.S + 2 Deamidated (
1707.9005	1706.8932	1706.8410	30.6	371	- 385	1	---	R.GIEYSRASPQPMISR.L + Oxidation (M
1707.9005	1706.8932	1706.8410	30.6	371	- 385	1	---	R.GIEYSRASPQPMISR.L + Oxidation (M
2705.3350	2704.3277	2704.2989	10.7	145	- 167	1	---	K.SVSPLVWCRQVLDYPSPDVECAK.K

No match to: 842.5737, 842.5737, 856.5821, 856.5821, 870.6026, 870.6026, 877.1173, 877.1173, 896.5347, 897.6122, 897.6122, 928.5591, 928.5591, 1051.6309, 1051.6309, 1259.7749, 1259.7749, 1285.7260, 1285.7260, 1297.7300, 1308.7545, 1308.7545, 1320.7034, 1323.7662, 1323.7662, 1331.8176, 1331.8176, 1374.7856, 1375.8502, 1375.8502, 1413.8215, 1419.8638, 1419.8638, 1434.8639, 1434.8639, 1493.8533, 1493.8533, 1505.8356, 1505.8356, 1551.8925, 1551.8925, 1638.9702, 1638.9702, 1791.8708, 1791.8709, 1994.1136, 2050.1382, 2384.1306, 2399.1768, 2717.2629

20. gi|149041536 **Mass:** 34338 **Score:** 30 **Expect:** 75 **Queries matched:** 5

rCG58022, isoform CRA_a [Rattus norvegicus]

Observed	Mr(expt)	Mr(calc)	ppm	Start	End	Miss	Ions	Peptide
1179.6890	1178.6817	1178.5931	75.1	240	- 248	1	24	R.YQEVLDKQR.Q + Deamidated (NQ)
1179.6890	1178.6817	1178.5931	75.2	240	- 248	1	---	R.YQEVLDKQR.Q + Deamidated (NQ)
1323.7662	1322.7589	1322.5772	137	143	- 153	1	---	K.QLASRNCSEEK.C + 2 Deamidated (NQ
1323.7662	1322.7590	1322.5772	137	143	- 153	1	---	K.QLASRNCSEEK.C + 2 Deamidated (NQ
1994.1136	1993.1063	1992.8946	106	46	- 63	0	---	K.NLSPPSVSSQMITEESNR.N + 2 Deamida

No match to: 842.5737, 842.5737, 856.5821, 856.5821, 868.5806, 870.6026, 870.6026, 877.1173, 877.1173, 896.5347, 897.6122, 897.6122, 928.5591, 928.5591, 1051.6309, 1051.6309, 1259.7749, 1259.7749, 1285.7260, 1285.7260, 1297.7300, 1308.7545, 1308.7545, 1320.7034, 1331.8176, 1331.8176, 1374.7856, 1375.8502, 1375.8502, 1413.8215, 1419.8638, 1419.8638, 1434.8639, 1434.8639, 1463.8888, 1463.8888, 1493.8533, 1493.8533, 1505.8356, 1505.8356, 1551.8925, 1551.8925, 1638.9702, 1638.9702, 1707.9005, 1707.9005, 1791.8708, 1791.8709, 2050.1382, 2384.1306, 2399.1768, 2705.3350, 2717.2629

Search Parameters

Type of search : Sequence Query
Enzyme : Trypsin
Fixed modifications : Carbamidomethyl (C)
Variable modifications : Deamidated (NQ),Oxidation (M)
Mass values : Monoisotopic
Protein Mass : Unrestricted
Peptide Mass Tolerance : ± 250 ppm
Fragment Mass Tolerance: ± 0.4 Da
Max Missed Cleavages : 1
Instrument type : MALDI-TOF-TOF
Query1 (842.5737,1+) : <no title>
Query2 (842.5737,1+) : Locus:1.G4.2.1.25
Query3 (856.5821,1+) : Locus:1.G4.2.1.18
Query4 (856.5821,1+) : <no title>
Query5 (868.5806,1+) : <no title>
Query6 (870.6026,1+) : Locus:1.G4.2.1.23
Query7 (870.6026,1+) : <no title>
Query8 (877.1173,1+) : <no title>
Query9 (877.1173,1+) : Locus:1.G4.2.1.12
Query10 (896.5347,1+) : <no title>
Query11 (897.6122,1+) : Locus:1.G4.2.1.22
Query12 (897.6122,1+) : <no title>

```
Query13 (928.5591,1+)  : Locus:1.G4.2.1.20
Query14 (928.5591,1+)  : <no title>
Query15 (1051.6309,1+) : Locus:1.G4.2.1.17
Query16 (1051.6309,1+) : <no title>
Query17 (1179.6890,1+) : Locus:1.G4.2.1.19
Query18 (1179.6890,1+) : <no title>
Query19 (1259.7749,1+) : Locus:1.G4.2.1.14
Query20 (1259.7749,1+) : <no title>
Query21 (1285.7260,1+) : Locus:1.G4.2.1.24
Query22 (1285.7260,1+) : <no title>
Query23 (1297.7300,1+) : <no title>
Query24 (1308.7545,1+) : <no title>
Query25 (1308.7545,1+) : Locus:1.G4.2.1.15
Query26 (1320.7034,1+) : <no title>
Query27 (1323.7662,1+) : <no title>
Query28 (1323.7662,1+) : Locus:1.G4.2.1.13
Query29 (1331.8176,1+) : <no title>
Query30 (1331.8176,1+) : Locus:1.G4.2.1.9
Query31 (1374.7856,1+) : <no title>
Query32 (1375.8502,1+) : <no title>
Query33 (1375.8502,1+) : Locus:1.G4.2.1.21
Query34 (1413.8215,1+) : <no title>
Query35 (1419.8638,1+) : Locus:1.G4.2.1.6
Query36 (1419.8638,1+) : <no title>
Query37 (1434.8639,1+) : Locus:1.G4.2.1.7
Query38 (1434.8639,1+) : <no title>
Query39 (1463.8888,1+) : Locus:1.G4.2.1.4
Query40 (1463.8888,1+) : <no title>
Query41 (1493.8533,1+) : Locus:1.G4.2.1.10
Query42 (1493.8533,1+) : <no title>
Query43 (1505.8356,1+) : Locus:1.G4.2.1.8
Query44 (1505.8356,1+) : <no title>
Query45 (1551.8925,1+) : Locus:1.G4.2.1.16
Query46 (1551.8925,1+) : <no title>
Query47 (1638.9702,1+) : <no title>
Query48 (1638.9702,1+) : Locus:1.G4.2.1.11
Query49 (1707.9005,1+) : <no title>
Query50 (1707.9005,1+) : Locus:1.G4.2.1.5
Query51 (1791.8708,1+) : <no title>
Query52 (1791.8709,1+) : Locus:1.G4.2.1.2
Query53 (1994.1136,1+) : <no title>
Query54 (2050.1382,1+) : <no title>
Query55 (2384.1306,1+) : <no title>
Query56 (2399.1768,1+) : <no title>
Query57 (2705.3350,1+) : <no title>
Query58 (2717.2629,1+) : <no title>
```

**Mascot:** http://www.matrixscience.com/
